# A phosphoinositide switch mediates exocyst recruitment to multivesicular endosomes for exosome secretion

**DOI:** 10.1038/s41467-023-42661-0

**Published:** 2023-10-28

**Authors:** Di-Ao Liu, Kai Tao, Bin Wu, Ziyan Yu, Malwina Szczepaniak, Matthew Rames, Changsong Yang, Tatyana Svitkina, Yueyao Zhu, Fengyuan Xu, Xiaolin Nan, Wei Guo

**Affiliations:** 1 Department of Biology, School of Arts & Sciences, University of Pennsylvania, Philadelphia, PA 19104 USA; 2 Program in Quantitative and Systems Biology, Department of Biomedical Engineering, Oregon Health and Science University, 2730 S. Moody Ave, Portland, OR 97201 USA; 3 Cancer Early Detection Advanced Research Center, Knight Cancer Institute, Oregon Health and Science University, 2720 S. Moody Ave., Portland, OR 97201 USA; 4 Department of Pathology & Laboratory Medicine, Children’s Hospital of Philadelphia Research Institute, Philadelphia, PA 19104 USA; 5 Department of Bioengineering, University of Pennsylvania, Philadelphia, PA 19104 USA

**Keywords:** Multivesicular bodies, Secretion

## Abstract

Exosomes are secreted to the extracellular milieu when multivesicular endosomes (MVEs) dock and fuse with the plasma membrane. However, MVEs are also known to fuse with lysosomes for degradation. How MVEs are directed to the plasma membrane for exosome secretion rather than to lysosomes is unclear. Here we report that a conversion of phosphatidylinositol-3-phosphate (PI(3)P) to phosphatidylinositol-4-phosphate (PI(4)P) catalyzed sequentially by Myotubularin 1 (MTM1) and phosphatidylinositol 4-kinase type IIα (PI4KIIα) on the surface of MVEs mediates the recruitment of the exocyst complex. The exocyst then targets the MVEs to the plasma membrane for exosome secretion. We further demonstrate that disrupting PI(4)P generation or exocyst function blocked exosomal secretion of Programmed death-ligand 1 (PD-L1), a key immune checkpoint protein in tumor cells, and led to its accumulation in lysosomes. Together, our study suggests that the PI(3)P to PI(4)P conversion on MVEs and the recruitment of the exocyst direct the exocytic trafficking of MVEs for exosome secretion.

## Abstract (web summary)

The molecular mechanism by which exosomes are released from cells is unclear. Here the authors report that a phosphatidylinositide conversion couples the recruitment of the octameric exocyst complex to multivesicular endosomes for exosome secretion.

## Introduction

Studies in recent years have established the role of extracellular vesicles (EVs), especially exosomes, as a highly effective means of cell-cell communication. Exosomes are implicated in a wide range of pathophysiological conditions from tumor immune evasion to neurodegeneration^1–7^. Despite the great interest in exosomes in different fields, basic cell biological understanding of exosome secretion is disproportionally lacking.

Exosomes originate from intraluminal vesicles (ILVs) present within multivesicular endosomes (MVEs). The Endosomal Sorting Complexes Responsible for Transport (ESCRT) recruit endocytosed cargos and mediate membrane invagination for the formation of the ILVs^1–9^. Subsequently, the MVEs either fuse with the lysosomes for degradation or are transported to the cell periphery, where they are tethered to and fuse with the plasma membrane to release the ILVs as exosomes. A central unanswered question in the field is: what are the molecules that direct MVEs to the plasma membrane for exosome secretion rather than to lysosomes for degradation? Although previous studies have established the important roles of the Rab family of small GTPases (e.g., Rab27 and Rab11) and the SNARE proteins in exosome secretion^10–14^, the molecular mechanism directing the trafficking of the MVEs to the plasma membrane for exosome secretion rather than lysosomal degradation remains unknown.

The exocyst is an octameric protein complex consisting of Sec3, Sec5, Sec6, Sec8, Sec10, Sec15, Exo70 and Exo84^15^. Works from the budding yeast *Saccharomyces cerevisiae* and mammalian cells have established that the exocyst targets and tethers *trans*-Golgi network (TGN)-derived secretory vesicles to the plasma membrane and promotes SNARE-mediated fusion^15–20^. Exocyst subunits interact with the SNARE proteins and with the Rab family of small GTPases such as Rab11 and Rab27 during conventional exocytosis^21,22^, but whether and how the exocyst mediates exosome secretion remains elusive.

In addition to the protein machinery, vesicular trafficking is also controlled by phosphoinositides^23–25^. While the endolysosomal compartments are marked by phosphatidylinositol-3-phosphate (PI(3)P) and phosphatidylinositol-3,5-bisphosphate (PI(3,5)P_2_) on their surface, exocytic trafficking often requires the generation of phosphatidylinositol-4-phosphate (PI(4)P)^26–30^. PI4-kinases, such as PI4KIIα and PI4KIIIβ, have been reported to localize to the endosomes and lysosomes, where they generate PI(4)P from phosphatidylinositol (PI)^28,31,32^. As exosomes are generated from endosomal compartments, we hypothesize that the generation of PI(4)P by PI4-kinases helps direct MVEs for exocytic trafficking.

Here we report that a PI(3)P to PI(4)P switch on MVEs mediates the recruitment of the exocyst for the exocytic trafficking of MVEs. This phosphoinositide switch is mediated sequentially by the PI(3)P phosphatase, MTM1, and the PI(4)P kinase, PI4KIIα. We further demonstrate that both PI(4)P generation and exocyst function are required for exosomal secretion rather than lysosomal distribution of PD-L1, a key immune checkpoint protein in tumor cells that mediates immune suppression.

## Results

### Inhibition of the exocyst proteins reduces exosome secretion

**Fig. 1:**
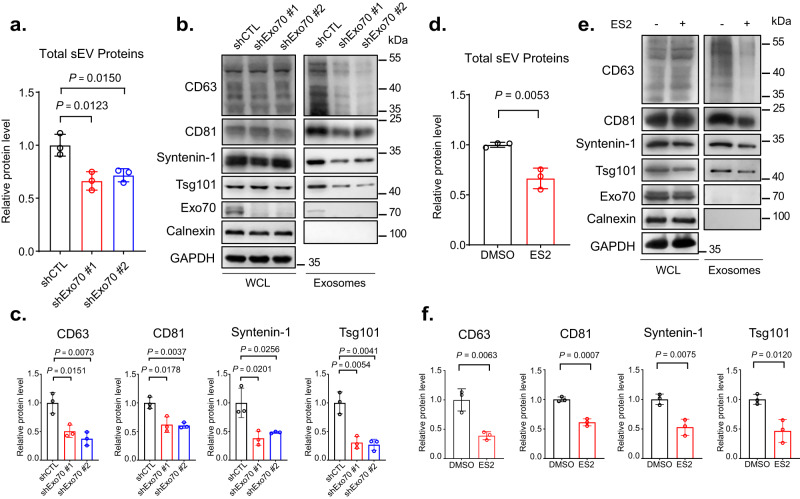
Exo70 regulates the secretion of exosomes. **a** Concentrations of total exosome proteins derived from culture supernatants of MDA-MB-231 cells stably expressing the control (“shCTL”) or Exo70 shRNAs. Two shRNA sequences targeting different regions of Exo70 sequence (“shRNA#1” and “shRNA#2”) were used (*n* = 3). The average concentration of exosome proteins from cells treated with Exo70 shRNAs was normalized to that of cells treated with shCTL. **b** Western blotting analysis of whole cell lysates (“WCL”) and exosomes isolated from the culture media of the control and Exo70 knockdown cells. Exosomes from equal amounts of cells (1 × 10^7^) were collected. The Exo70 and exosome marker proteins CD63, CD81, Syntenin-1, and Tsg101, and the ER marker protein Calnexin are examined. GAPDH is used as a loading control for whole cell lysates. **c** Quantification of the amounts of CD63, CD81, Syntenin-1, and Tsg101 on exosomes derived from the control and Exo70 knockdown cells (*n* = 3). The average levels of each protein from cells expressing Exo70 shRNAs are normalized to that from shCTL cells. **d** Quantification of total exosome proteins derived from DMSO or ES2-treated cells (*n* = 3). **e** Western blot analysis of whole cell lysates and exosomes isolated from DMSO and ES2-treated cells. The average concentration of exosome proteins from cells treated with ES2 is normalized to that of cells treated with DMSO. **f** Quantification of CD63, CD81, Syntenin-1, and Tsg101 on exosomes from DMSO and ES2-treated cells (*n* = 3). The average level of each exosomal protein from cells treated with ES2 is normalized to that of cells treated with DMSO. Data are presented as mean ± s.d. of three independent biological replicates. *P*-values are calculated using two-sided unpaired *t*-test.

To understand the molecular mechanism that directs MVE trafficking to the plasma membrane, we first determined the function of the exocyst in exosome secretion. We knocked down the exocyst subunit Exo70 in MDA-MB-231 cells by the shRNA. Exosomes derived from the control (“shCTL”, scrambled shRNA sequence) and knockdown (shEox70 #1 and #2) cells were isolated from conditioned media by differential centrifugation as previously described^33^. The diameters and morphology of isolated exosomes were verified by nanoparticle tracking analysis (NTA) (Supplementary Fig. 1a) and transmission electron microscopy (TEM) (Supplementary Fig. 1b). Cells with Exo70 knockdown showed lower levels of exosome secretion as demonstrated by the reduced amounts of total exosomal proteins isolated from the cell culture media (Fig. 1a), as well as by western blot analysis of the exosomal marker proteins CD63, CD81, Syntenin-1 and Tsg101 (Fig. 1b, c). NTA also showed a decrease in the number of exosomes isolated from Exo70 knockdown cells (Supplementary Fig. 1c). In addition to Exo70 knockdown, cells expressing shRNAs targeting other exocyst components, Sec8, Sec10, or Exo84, also exhibited reduced exosome secretion (Supplementary Fig. 1d–h). Besides shRNA-mediated knockdown, we also treated cells with Endosidine-2 (ES2), a chemical inhibitor of Exo70^34^. Compared with the vehicle (DMSO) treated cells, exosome secretion from the ES2-treated cells was reduced (Fig. 1d–f). In addition to MDA-MB-231 cells, treatment of COS-7 or PANC-1 cells with Exo70 shRNAs or with ES2 also decreased exosome secretion (Supplementary Fig. 2).

### Inhibition of the exocyst proteins leads to intracellular accumulation of MVEs

**Fig. 2:**
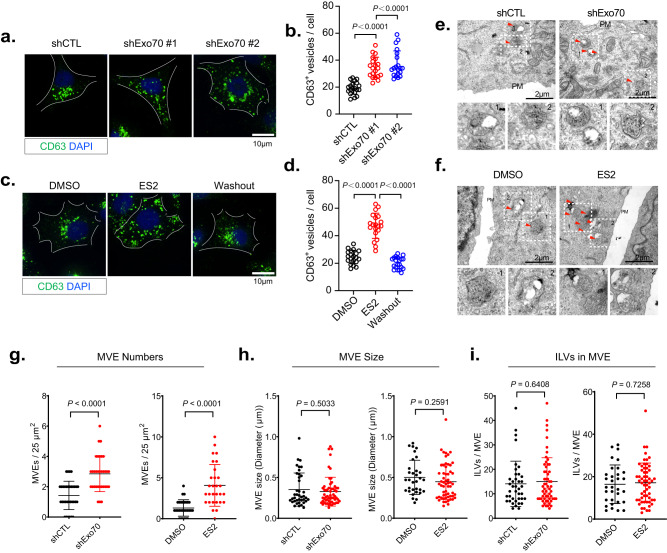
Inhibition of Exo70 leads to intracellular accumulation of MVEs. **a** Immunofluorescence staining of CD63 in cells stably expressing the control or Exo70 shRNAs. Scale bar = 10 μm. **b** Quantification of MVEs marked by CD63 in cells stably expressing the control or Exo70 shRNAs (*n* = 20 cells). Data are presented as mean ± s.d. *P*-values are calculated using two-sided unpaired *t*-test. **c** Immunofluorescence staining of CD63 in cells treated with DMSO, ES2, or after treatment and washout of ES2. Scale bar = 10 μm. Data are presented as mean ± s.d. *P*-values are calculated using two-sided unpaired *t*-test. **d** Quantification of CD63-positive MVEs in cells treated with DMSO, ES2, or after the treatment and washout of ES2 (*n* = 20 cells). Data are presented as mean ± s.d. *P*-values are calculated using two-sided unpaired *t*-test. **e**, **f** Representative electron microscopy images of MVEs in cells stably expressing control or Exo70 shRNA (**e**) or in cells treated with DMSO or ES2 (**f**). Scale bars = 2 μm. Red arrows indicate MVEs. Dashed rectangles indicate regions zoomed in the respective lower panels. **g**–**i** Quantification of the number (**g**) and size (**h**) of MVEs and the ILV numbers of each MVE (**i**) in EM images in conditions shown in (**e**) (*n* = 32 cells for shCTL/shExo70, *n* = 29 cells for DMSO/ES2). Data are presented as mean ± s.d. *P*-values are calculated using two-sided unpaired *t*-test.

To test whether the decrease in exosome secretion from the Exo70 deficient cells is a result of defective MVE trafficking to the plasma membrane, we examined MVEs in MDA-MB-231 cells by immunostaining of CD63. Exo70 knockdown led to an increase of CD63-positive MVEs inside the cells (Fig. 2a, b). ES2 treatment also led to intracellular accumulation of CD63-positive MVEs in cells, which disappeared after ES2 washout (Fig. 2c, d). In addition to MDA-MB-231 cells, we also observed the accumulation of CD63 marked MVEs inside COS-7 or PANC-1 treated with Exo70 shRNAs or ES2 (Supplementary Fig. 3). To unambiguously identify MVEs, we examined cells by electron microscopy. Compared with their respective control cells, the Exo70 knockdown cells or cells treated with ES2 exhibited increased accumulation of MVEs, which are characterized by the presence of ILVs (Fig. 2e, f). Quantification of the EM images showed a significant increase in the number of MVEs in Exo70 knockdown cells or cells after ES2 treatment (Fig. 2g), whereas the size of MVEs, or ILVs inside each MVE, did not show a significant difference relative to the control cells (Fig. 2h, i).

**Fig. 3:**
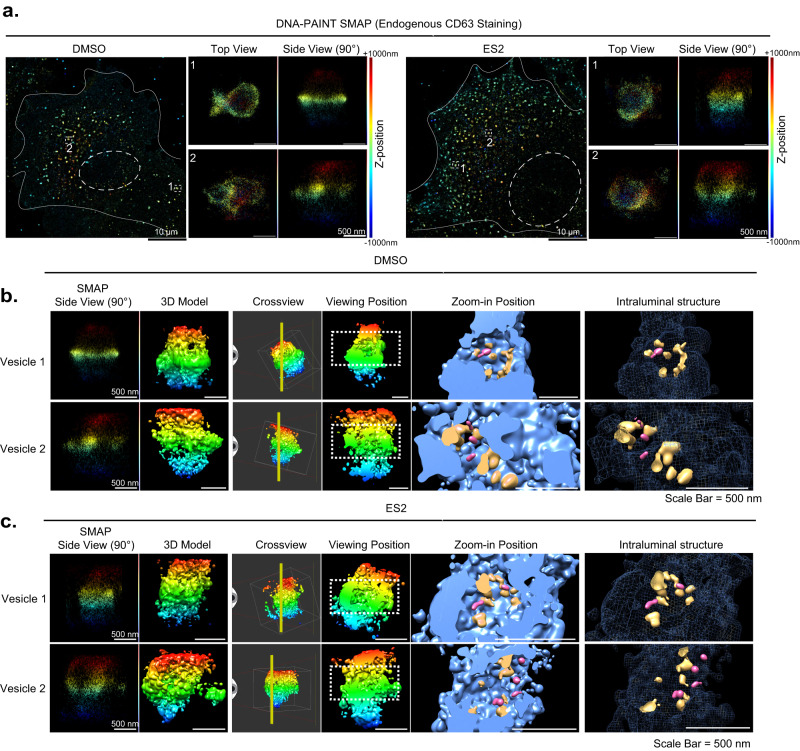
Super-resolution imaging of MVEs by DNA-PAINT. **a** 3D DNA-PAINT images of COS-7 cells treated with DMSO (left panels) or ES2 (right panels). The relative z-positions of the localizations within the ~2 µm axial range (effective imaging range is ~1.5 µm) are color-coded as indicated. The white dashed rectangles mark the CD63-positive vesicles (zoomed in) and are shown as viewed from the apical side of the cells (top view) or from an angle perpendicular to the bottom membrane (side view). **b**, **c** Volumetric renderings of the CD63-positive vesicles shown in (**a**) from DMSO-treated (**b**) and ES2-treated (**c**) cells. The 3D localizations are converted into molmaps using UCSF Chimera with a sigma factor of 1.5, which results in a final rendering resolution of ~24 nm. Left-most columns are side views from SMAP, each paired with a corresponding 3D structural model from Chimera (2nd columns). The vertical yellow line in “Crossview” panel (third columns) corresponds to the cutting plane for the subsequent cross-section views (columns four, five, and six), all shown as viewed from the left of the cutting plane. White boxes in the cross views indicate the outer bounds of SMAP localizations, while the dotted white rectangles in the viewing position (fourth columns) indicate the border of the zoom-in views, shown as filled volumes (fifth columns) or wireframes (sixth columns). Within both zoom-in views, pink-colored structures indicate intraluminal vesicles, which correspond to densities within the interior cavity that are detached from the enclosing lumen. The orange-colored structures indicate potential invaginations that are budding off and still connected to the enclosing MVE membrane. Scale bars = 10 µm (white, as in overviews) or 500 nm (white, as in close-up views). All experiments were repeated more than three times in (**a,** **b** and **c**).

We also examined the MVEs by 3D super-resolution imaging using DNA-PAINT^35,36^, which is a single-molecule localization microscopy (SMLM) technique that offers spatial resolution below the diffraction limit of conventional light microscopy. In combination with optical astigmatism^37^, 3D super-resolution imaging would allow us to observe CD63-positive MVEs at a nanoscale. For these experiments, a highly inclined and laminated optical (HiLo) illumination scheme was used in order to probe ~1.5 µm into the intracellular space, rather than only 100–200 nm as in total internal reflection fluorescence (TIRF) microscopy. We chose to use COS-7 cells for 3D imaging because of their flatness so that raw DNA-PAINT images could be acquired at a high signal-to-background ratio. In this experiment, COS-7 cells were treated with DMSO or ES2, fixed, and stained with an anti-CD63 primary antibody and a DNA-conjugated secondary antibody for DNA-PAINT imaging. We observed that ES2 treatment led to the accumulation of CD63^+^ vesicles in COS-7 cells in the super-resolution images reconstructed using SMAP (Super-resolution Microscopy Analysis Platform)^38^ (Fig. 3a). In both control and ES2-treated cells, we were able to obtain 3D structures of CD63^+^ vesicles (Fig. 3a–c). To better inspect their internal structures, we exported the 3D coordinates and generated volumetric images in Chimera, an open-source visualization and analysis software suite^39^. The resulting volumetric renderings clearly demonstrated the overall structure of the CD63^+^ vesicles. Cut-through views (rightmost panels, Fig. 3b, c) showed that, while the perimeters of these vesicles are largely closed, their internal spaces are often occupied by smaller CD63^+^ vesicles indicative of ILVs (Fig. 3b, c). The enclosing vesicles were typically 1–2 µm in diameter and the ILVs were typically ~100 nm (Fig. 3a–c). Of note, even the smaller vesicles were well above our resolution limit for the labeled structure (~20 nm lateral and ~40 nm axial). While some of the ILVs (pink structures in the rightmost panels of Fig. 3b, c) were located inward, many appeared attached to the enclosing membrane (orange structures in the same image panels). Some of the attachment could be due to a lack of clear resolution of enclosing membrane from ILV membrane located in close proximity. We also noticed that some of the structures are likely invaginations of the limiting membrane (Fig. 3b, c, Supplementary Movie 1 and Movie 2). The presence of these different types of intraluminal structures implies a dynamic process that constantly reshapes the overall morphology as well as the internal structure of the MVEs. We did not observe major differences in the 3D structures of MVEs between the control and ES2-treated cells, although potential structural changes should not be excluded.

### The exocyst associates with MVEs

**Fig. 4:**
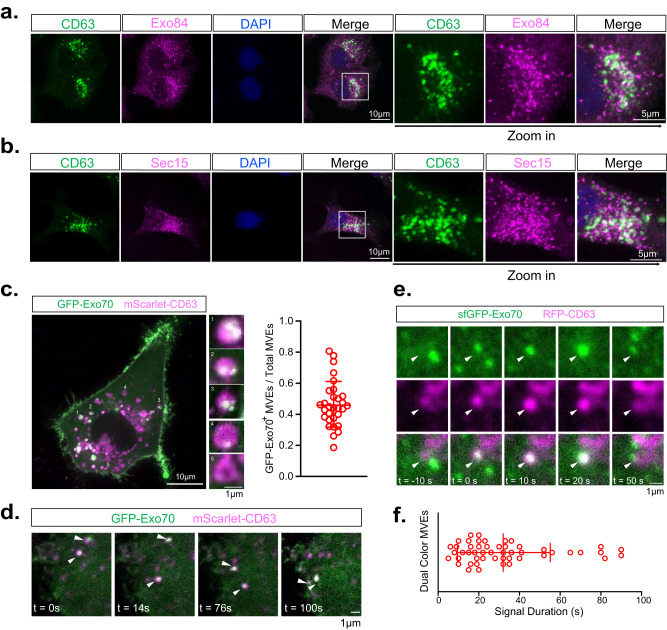
The exocyst is associated with a subpopulation of MVEs. **a**, **b** Immunostaining of CD63 and Exo84 (**a**) and CD63 and Sec15 (**b**) in MDA-MB-231 cells. Scale bar = 10 µm (overview) or 5 µm (zoom-in view). (*n* = 12 cells) and quantification is presented in Supplementary Fig. 4. **c** Confocal imaging of mScarlet-CD63^+^ and GFP-Exo70^+^ vesicles. Five representative CD63^+^ vesicles are enlarged in small panels. Quantification of the portion of mScarlet-CD63^+^ GFP-Exo70^+^ vesicles in total mScarlet-CD63^+^ vesicles is shown at right (*n* = 30 cells). Data are presented as mean ± s.d. Scale bar = 10 µm (overview) or 1 µm (zoom-in view). **d** Time-lapse confocal imaging of mScarlet-CD63^+^ and GFP-Exo70^+^ vesicles (arrowhead) moving toward the plasma membrane. Scale bar = 1 μm. **e** TIRF microscopy imaging of an RFP-CD63^+^ and sfGFP-Exo70^+^ vesicle (arrowhead) during its arrival at the plasma membrane. Scale bar = 1 μm. Scale bar = 1 μm. **f** Quantification of the duration of the RFP-CD63^+^ and sfGFP-Exo70^+^ vesicle at the plasma membrane in (**e**) (*n* = 50 vesicles). Data are presented as mean ± s.d.

Next, we examined the potential association of the exocyst subunits with MVEs. To this end, we performed immunofluorescence microscopy of endogenous proteins in MDA-MB-231 cells using antibodies against the MVE marker CD63 and the exocyst subunits Sec15 and Exo84. We found that Sec15 and Exo84 partially colocalized with CD63 (Fig. 4a, b), but not the early endosome marker EEA1 (Supplementary Fig. 4a, b). Sec15 and Exo84 localized to discrete regions of CD63-positive MVEs as indicated by their overlapping signals (Supplementary Fig. 4c) and colocalization analysis were performed for images across all z-stacks (also see “Methods”). The exocyst is localized to the surface of MVEs; this is different from the fluorescence signals of CD63, which, as a cargo protein, is localized to both the surface and the intraluminal vesicles of MVEs. This localization pattern is consistent with the role of the exocyst in MVE tethering as discussed later.

Not all the exocyst colocalized with MVEs (Fig. 4a, b; Supplementary Fig. 4c). This is as expected as there are different subpopulations of MVEs that are destined either to the plasma membrane (“secretory MVEs”) or to the lysosomes (“degradative MVEs”); the exocyst may not associate with all subpopulations of MVEs. Moreover, we expect that the exocyst also associates with other secretory structures such as those originating from TGN or recycling endosomes.

To track the exocyst on MVEs in live cells, we performed confocal microscopy imaging of cells expressing mScarlet-CD63 and GFP-Exo70. We observed colocalization of these two proteins on a subpopulation of MVEs (Fig. 4c), consistent with the above immunofluorescence data from fixed cells. Time-lapse imaging showed that CD63 and Exo70 were co-transported on the same vesicles (Fig. 4d) (Supplementary Movie 3). To track Exo70-associated MVEs specifically near the plasma membrane, we performed time-lapse TIRF microscopy using cells with their endogenous Exo70 tagged with sfGFP^40^. We observed the co-appearance of sfGFP-Exo70 and RFP-CD63 near the plasma membrane and their subsequent disappearance, which probably resulted from MVE fusion with the plasma membrane (Supplementary Movie 4) (Fig. 4e)^40,41^. The signal duration of vesicles at the plasma membrane was slightly longer for the MVEs (Fig. 4f) than that of conventional secretory vesicles^40,41^.

### The exocyst directs the trafficking of MVEs

Previous studies suggested that the exocyst targets TGN-derived secretory vesicles to the plasma membrane for exocytosis^18,19,42^. Our finding that the exocyst associates with MVEs suggests that the exocyst also directs MVEs to the plasma membrane for the release of exosomes. To test this hypothesis, we adopted an ectopic targeting strategy to test whether artificially targeting the exocyst to mitochondria would redirect MVEs to mitochondria. Since the plasma membrane-specific t-SNARE proteins are not present on mitochondria, the redirected vesicles would be expected to accumulate at mitochondria, but not fuse with them. We previously showed in yeast cells that mitochondria ectopic targeting of the exocyst subunit Sec3, the most plasma membrane proximal component of the exocyst complex, redirected the other exocyst subunits and TGN-derived secretory vesicles from the plasma membrane to mitochondria^43^. This ectopic targeting strategy has also been used to study vesicle tethering at the Golgi apparatus^44–46^.

**Fig. 5:**
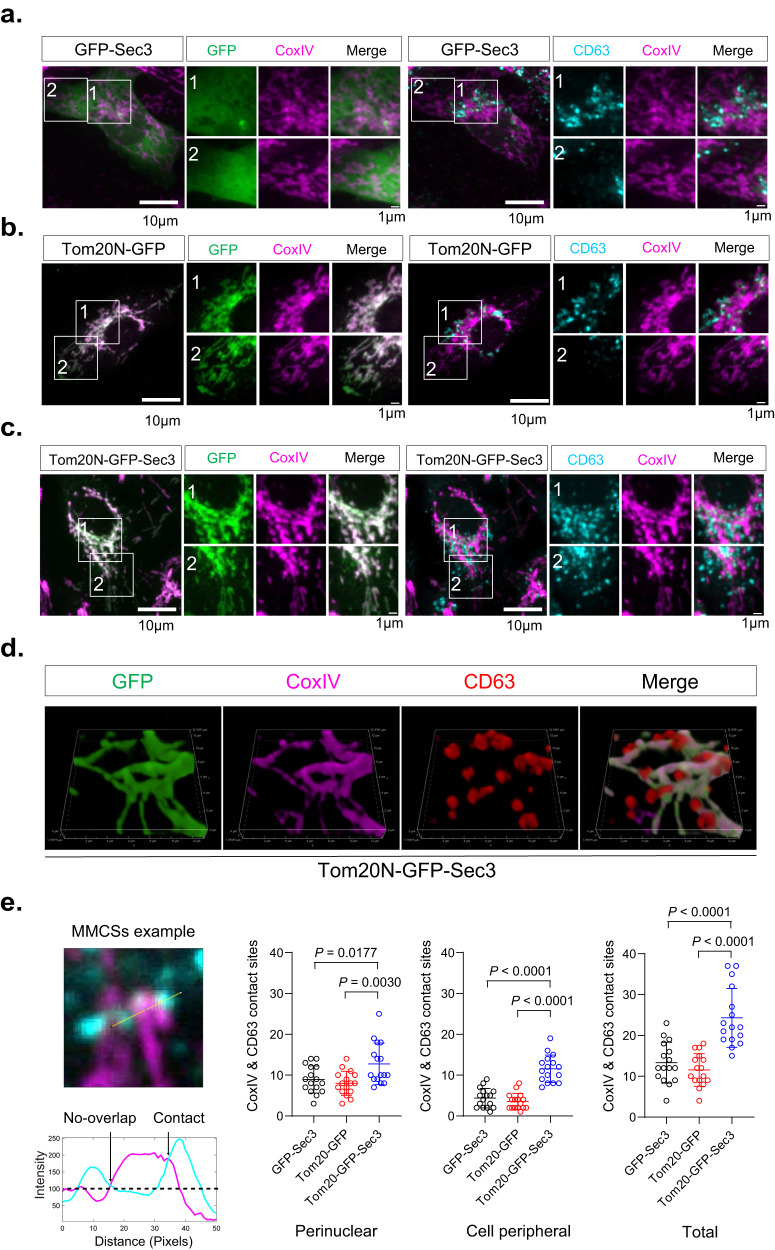
Ectopic recruitment of MVEs to mitochondria by Tom20N-GFP-Sec3. **a** GFP-Sec3 (green) shows diffuse cytosolic distribution and does not colocalize with mitochondria marked by CoxIV (magenta). CD63 (cyan) is mainly present in the perinuclear region (marked in Box 1), and to a lesser extent, in the peripheral region (Box 2) of the cell. These two regions are magnified at right. **b** Tom20N-GFP is localized to mitochondria. CD63 is mainly present in the perinuclear region, and to a lesser extent, in the peripheral region of the cell. **c** Tom20N-GFP-Sec3 is localized to mitochondria. CD63-positive MVEs (cyan) are also recruited to mitochondria in these cells and are present in both the perinuclear and peripheral cell regions. **d** 3D reconstruction of a representative cell expressing Tom20N-GFP-Sec3 shows the association of CD63-positive vesicles with mitochondria in a pattern that resembles “tomatoes on the vine”. **e** A representative image of MMCS is shown at the left. The intensity profile shows one contact site with an overlapped CoxIV-CD63 signal above the background signal. Quantification of CD63-CoxIV contact sites (see “Methods”) in the perinuclear region, peripheral region, and the whole cell (*n* = 15 cells) are shown at the right. Scale bars are indicated in the panels. Data are presented as mean ± s.d. *P*-values are calculated using two-sided unpaired *t*-test.

Here, GFP-Sec3 was used as a non-ectopic targeting control (Fig. 5a). To create ectopically located proteins to mitochondria, we fused the N-terminal sequence of the mitochondrial outer membrane protein Tom20 (amino acids 1-47; “Tom20N”) to GFP (“Tom20N-GFP”). When expressed in cells, Tom20N-GFP colocalized with the mitochondria marker CoxIV (Fig. 5b, left panel), indicating successful ectopic targeting. We then fused Tom20N to GFP-Sec3 (“Tom20N-GFP-Sec3”). The resulting fusion protein is also localized to mitochondria (Fig. 5c, left panel).

In cells expressing Tom20N-GFP-Sec3, but not those expressing GFP-Sec3, endogenous Exo84 were targeted to the mitochondria, as indicated by their appearance along the CoxIV-labeled mitochondrial network (Supplementary Fig. 5a, b). Previous studies of the molecular assembly of the exocyst complex in both yeast and mammalian cells showed that Exo84 is in a different subcomplex (“subcomplex II”) than Sec3 (a subunit of “subcomplex I”)^15,40,47,48^. The recruitment of Exo84 to mitochondria in cells expressing Tom20N-GFP-Sec3 suggests that the exocyst subunits ectopically assemble at mitochondria in these cells.

Next, we examined the distribution of MVEs using CD63 staining. MVEs were mostly distributed at the perinuclear regions in cells expressing Tom20N-GFP or GFP-Sec3 (Fig. 5a, b, right panel). In cells expressing Tom20N-GFP-Sec3, however, MVEs were re-distributed to mitochondria marked by CoxIV (Fig. 5c, right panel). 3D rendering showed a clearer MVE-mitochondria association that resembles “tomatoes on the vine” (Fig. 5d), while the two control groups (Tom20N-GFP and GFP-Sec3) did not show such an association. We next quantified the mitochondrion-MVE contact sites (MMCS) at both the perinuclear region and the cell peripheral region in cells by overlapping fluorescence signals of CoxIV and CD63 using a method adapted from the study of the ER-endosome contact sites^49^. The number of MMCSs in cells expressing Tom20N-GFP-Sec3 was significantly higher in comparison to the control cells in both regions (Fig. 5e). In addition to CD63, we examined in this system the distribution of endogenous Rab27a, which regulates MVEs trafficking to the plasma membrane for exosome secretion^10^. Similar to CD63, we observed an association of Rab27a-positive vesicles with mitochondria in cells expressing Tom20N-GFP-Sec3 but not in cells expressing GFP-Sec3 (Supplementary Fig. 5c, d). To better visualize the association of MVEs with mitochondria, we performed EM experiments. The mitochondrion-MVE contacts were observed in cells expressing Tom20N-GFP-Sec3 (Supplementary Fig. 5e). 3D fluorescence image reconstruction clearly shows the association of mScarlet-CD63-positive MVEs and Exo84 with Tom20N-GFP-Sec3 positive mitochondria (Supplementary Fig. 5f). Furthermore, shRNA knockdown of Exo70 significantly reduced the number of MMCSs in Tom20N-GFP-Sec3 expressing cells (Supplementary Fig. 5g). The data provide further support that the exocyst is required for the ectopic targeting of the MVEs to mitochondria.

### A PI(3)P to PI(4)P conversion mediates the recruitment of the exocyst to MVEs

**Fig. 6:**
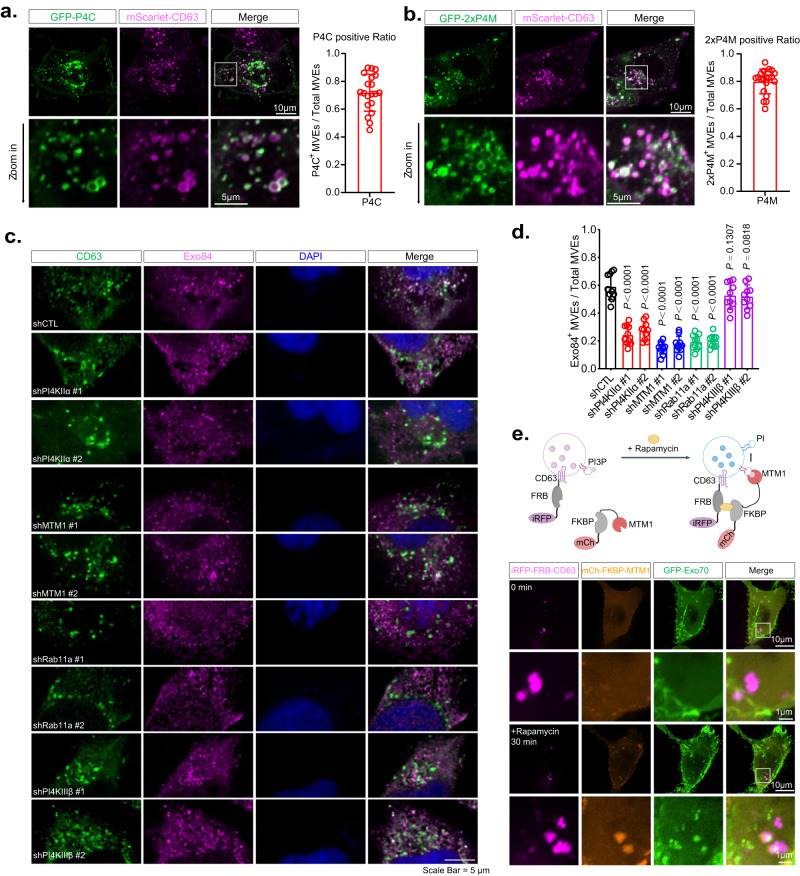
The PI(3)P to PI(4)P conversion controlled by MTM1 and PI4KIIα mediates the recruitment of the exocyst to the MVEs. **a**, **b** PI(4)P reporters GFP-P4C (**a**) and GFP-2xP4M (**b**) partially colocalize with mScarlet-CD63-positive MVEs. Zoom-in images of the boxed region are shown on the lower panels. Quantifications of the subpopulation of PI(4)P containing MVEs are shown at right (*n* = 20 cells). Scale bars are indicated in the panels. Data are presented as mean ± s.d. *P*-values are calculated using two-sided unpaired *t*-test. **c** Immunostaining of Exo84 (magenta) and CD63 (green) in cells stably expressing non-targeting shRNA (shCTL) or shRNAs targeting PI4KIIα, MTM1, Rab11a, or PI4KIIIβ. The nuclei are stained with DAPI. Scale bars = 5 µm. **d** Quantification of the subpopulation of Exo84-positive MVEs in cells with control shRNA or shRNAs targeting PI4KIIα, MTM1, Rab11a, or PI4KIIIβ (*n* = 10 cells). Data are presented as mean ± s.d. *P*-values are calculated using two-sided unpaired *t*-test. **e** Recruitment of Exo70 to CD63-positive MVEs by rapamycin induced MTM1 dephosphorylation of PI(3)P on MVEs. Upper panel: diagram of the experimental design. When rapamycin is added to cells co-expressing iRFP-FRB-CD63, mCherry-FKBP-MTM1, and GFP-Exo70, it induces the recruitment of MTM1 to MVEs, which leads to the conversion of PI(3)P to PI, thus providing a substrate for PI(4)P generation and subsequent exocyst recruitment. Lower panel: fluorescence microscopy imaging shows that both MTM1 and GFP-Exo70 associate with CD63-positive MVEs (arrowheads) 30 min after the addition of rapamycin. Scale bar = 1 μm. Experiments are performed more than three times.

Phosphoinositides localize to different intracellular membrane compartments and play a pivotal role in the specificity of membrane trafficking^23–25^. PI(4)P is mostly present on exocytic carriers, whereas PI(3)P and PI(3,5)P_2_ are characteristic of endolysosomal structures^23–26,50,51^. MVEs were reported to be marked by PI(3,5)P_2_ and previously considered as degradative organelles^27,52^. However, as MVEs can also be transported to the plasma membrane for exosome secretion (“secretory MVEs”), we reason that a subpopulation of MVEs destined for exosome secretion may carry PI(4)P on their surface. To test this hypothesis, we expressed previously well-characterized PI(4)P reporters, GFP-P4C or GFP-2xP4M^30^, to mark the PI(4)P positive membranes in MDA-MB-231 cells. Indeed, we observed colocalization of GFP-P4C or GFP-2xP4M with mScarlet-CD63 in cells (Fig. 6a, b). We also observed structures that were positive for PI(4)P only (Fig. 6a, b). They probably represent recycling endosomes and exocytic vesicles, such as those generated from the TGN^30^. Time-lapse live-cell imaging showed that GFP-P4C positive MVEs traffic to the plasma membrane (Supplementary Movie 5). We also examined the PI(3)P-positive MVEs using GFP-FYVE and found that approximately 45% of the total CD63-positive MVE population are positive for GFP-FYVE (Supplementary Fig. 6a, b). Exo70 and Sec3 were previously shown to bind to PI(4,5)P_2_ and mediate the exocyst association with the plasma membrane^53–56^. Using the GFP-PLCδ-PH, the PI(4,5)P_2_ biosensor^57^, we detected the PI(4,5)P_2_ signal on the plasma membrane, but not MVEs (Supplementary Fig. 6c).

Phosphatidylinositol-4 kinases (PI4Ks) mediate the generation of PI(4)P on the membrane, and PI4KIIα and PI4KIIIβ are the two candidate kinases that were reported to generate PI(4)P from PI on endosomes^28,32,58^. We therefore examined the potential colocalization of PI4KIIα or PI4KIIIβ with MVEs by immunofluorescence microscopy. Strong PI4KIIα signals were detected on MVEs marked by CD63 (Supplementary Fig. 7a). In contrast, PI4KIIIβ was mostly localized to the Golgi apparatus, nucleus, and some vesicles, consistent with previous reports^59^ (Supplementary Fig. 7b), but much less to CD63^+^ vesicles (Supplementary Fig. 7c). These results suggest that PI4KIIα is the potential PI4-kinase that generates PI(4)P on MVEs for exosome secretion. Supporting this notion, knocking down PI4KIIα, but not PI4KIIIβ (Supplementary Fig. 7d), decreased the association of Exo84 with CD63-positive vesicles (Fig. 6c, d). The loss of Exo84 from MVEs was a result of decreased PI4P level caused by the knockdown of PI4KIIα, as expression of wild-type Flag-tagged PI4KIIα in shPI4KIIα-expressing cells (the shRNA targets the 3’ UTR) restored the association of Exo84 with CD63-positive MVEs; expression of a kinase-dead PI4KIIα mutant (Flag-PI4KIIα-D308A) failed to rescue (Supplementary Fig. 7f, g). These results suggest that PI(4)P generated by PI4KIIα is needed for exocyst recruitment to MVEs. In addition to PI4KIIα, a reduced association of Exo84 with MVEs was also found in cells with knockdown of Rab11a (Fig. 6c, d), which was previously implicated in regulating exosome trafficking^14^.

Endosomes are often enriched with PI(3)P. To allow for the subsequent production of PI(4)P by PI4KIIa, it is first necessary to hydrolyze PI(3)P to PI by the PI(3)P phosphatase, MTM1^28,29^. We knocked down MTM1 in cells (Supplementary Fig. 7e) and observed decreased localization of Exo84 to CD63^+^ MVEs in these cells (Fig. 6c, d). To directly test whether the recruitment of MTM1 to CD63^+^ MVEs is required for exocyst recruitment, we adopted a rapid protein recruitment strategy previously developed by Balla and colleagues^30^. In MDA-MB-231 cells, we co-expressed MTM1 fused with mCherry and the 12-kDa FK506 Binding Protein (mCherry-FKBP-MTM1) and CD63 tagged with infrared fluorescent protein and FKBP12-Rapamycin Binding domain of mTOR (iRFP-FRB-CD63). Upon the addition of rapamycin, mCherry-FKBP-MTM1 would be recruited to MVEs marked by iRFP-FRB-CD63 due to the rapamycin-mediated interaction between FKBP and FRB. With MTM1 and PI4KIIα mediating the PI(3)P to PI(4)P transition on these MVEs, we would expect the exocyst to be recruited to these iRFP-FRB-CD63^+^ vesicles that previously had no exocyst or MTM1. Consistent with our expectation, the addition of rapamycin led to the recruitment of mCherry-FKBP-MTM1 to iRFP-FRB-CD63^+^ MVEs in cells, and GFP-Exo70 was recruited to these MVEs (Fig. 6e). This result further supports the role of MTM1 in the recruitment of exocyst to the MVEs for exosome secretion. Consistent with the notion, knockdown of MTM1 or PI4KIIα led to decreased PI(4)P level and increased PI(3)P level on MVEs (Supplementary Fig. 7h). Expression of PI4KIIα did not rescue the decrease of PI(4)P on MVEs in MTM1 knockdown cells (Supplementary Fig. 7i), supporting the sequential action of MTM1 and PI4KIIα in PI(4)P generation.

### Inhibition of exocytic trafficking of MVEs shift PD-L1 localization to lysosomes

To further test whether the exocyst/PI(4)P pathway affects exosome secretion, we investigated the impact of impairing this pathway on another exosome cargo, the immune checkpoint protein PD-L1. We and others recently found that PD-L1 is secreted on tumor-derived exosomes and that exosomal PD-L1 interacts with PD-1 on CD8 T cells, thereby mediating immune suppression^33,60–62^. PD-L1 can also be transported to lysosomes for degradation^63–65^. Here we use PD-L1 as a cargo protein to further examine the roles of exocyst and PI(4)P in MVE trafficking.

**Fig. 7:**
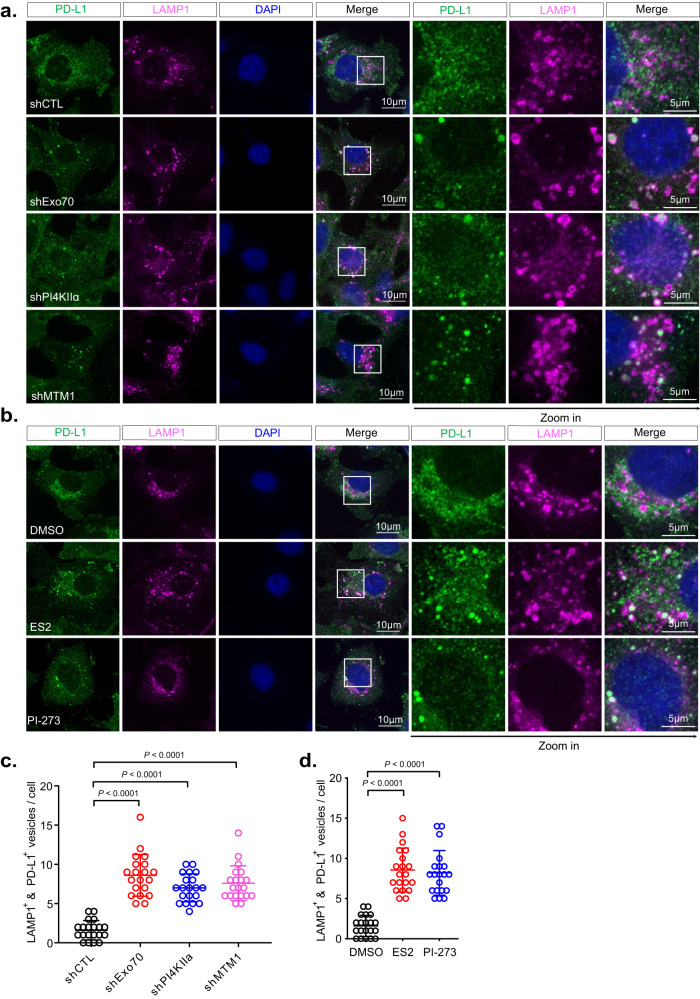
Inhibition of Exo70, PI4KIIα, or MTM1 leads to re-distribution of PD-L1 to the lysosomes. **a** Confocal imaging of PD-L1 in cells stably expressing control shRNA or shRNAs targeting Exo70, PI4KIIα, or MTM1. LAMP1 is used as a lysosome marker. Zoom-in images of the boxed regions are shown on the right. **b** Confocal imaging of PD-L1 in cells treated with DMSO, ES2, or PI-273. Zoom-in images of the boxed regions are shown to the right. **c** Quantification of PD-L1 and LAMP1 double positive vesicles in cells treated with control shRNA or shRNAs targeting Exo70, PI4KIIα, or MTM1 (*n* = 20 cells). Data are presented as mean ± s.d. *P*-values are calculated using two-sided unpaired *t*-test. **d** Quantification of PD-L1 and LAMP1 double positive vesicles in cells treated with DMSO, ES2, or PI-273 (*n* = 20 cells). Scale bars are indicated in the panels. Data are presented as mean ± s.d. *P*-values are calculated using two-sided unpaired *t*-test.

Using confocal immunofluorescence microscopy, we detected endogenous PD-L1 on intracellular membrane structures and the plasma membrane in MDA-MB-231 cells (Fig. 7a and Supplementary Fig. 8a). shRNAs knockdown of Exo70, PI4KIIα, or MTM1 led to a clear shift of PD-L1 distribution from Rab11 positive structures to LAMP1 lysosomes, which become abundant in the knockdown cells (Fig. 7a, c, Supplementary Fig. 8a, c). Similar observations were obtained in cells treated with ES2 that inhibits Exo70, or PI-273 that specifically inhibits PI4KIIα^66^ (Fig. 7b, d, Supplementary Fig. 8b, d). The shift in these knockdown cells can be rescued by expressing shRNA-resistant respective constructs (Supplementary Fig. 9).

In addition to examining PD-L1 distribution in cells by fluorescence imaging, we also assayed PD-L1 on exosomes isolated from the culture media. Lower levels of PD-L1 were detected on exosomes derived from cells treated with ES2 (Supplementary Fig. 10a, b) or the PI4KIIα inhibitor, PI-273 (Supplementary Fig. 10c, d). Together these results suggest that PD-L1 is transported to lysosomes when exosomal PD-L1 secretion is blocked.

## Discussion

For the secretion of exosomes, MVEs need to be directed to the cell periphery, where they dock and fuse with the plasma membrane. A mechanistic understanding of exosome secretion will thus require the identification of the molecules mediating the targeting of the MVEs to the plasma membrane rather than to the lysosomes, which, in many cases, were thought to be the default destination of the ILVs. Previous studies, especially those carried out in yeast, have established the role of the exocyst complex in the targeting and tethering of TGN-derived secretory vesicles to the plasma membrane for exocytosis^15,18–20^. However, the role of the exocyst in MVE targeting to the plasma membrane has not been established. A previous study in *C. elegans* epidermal cells ruled out the role of the exocyst in exosome secretion, although *sec-8* (worm homolog of human Sec8) knockdown by RNAi showed MVE accumulation^67^. On the other hand, it is interesting to note that the exocyst subunit Sec15 interacts with the GTP-bound form of Rab11 and Rab27^21,22^, which are both regulators of exosome secretion^10,14^. In addition, a previous study in kidney cells showed that inhibition of EXOC5 (Sec10) led to shorter primary cilia at the apical surface and reduced shedding of extracellular vesicles^68^. Recently, a study in head and neck cancer cells suggested that the exocyst might be involved in exosome secretion^69^.

Here we systematically studied the role of the exocyst in exosome secretion using a series of biochemical and imaging approaches. We showed that the exocyst subunits partially colocalized with the CD63-positive MVEs, often appearing at discrete sites on their surface. Disrupting the function of the exocyst, both by shRNA knockdown and chemical inhibition, led to reduced exosome secretion to the culture media and concomitant accumulation of MVEs in cells. Further live-cell imaging showed that Exo70 was associated with CD63-positive MVEs en route to the plasma membrane.

An important step toward understanding the secretion of exosomes is to determine the internal organization of MVEs. This has been challenging due to the small size of the enclosed ILVs, which are a defining feature of MVEs but are usually no more than 200 nm in diameter. This has precluded the use of confocal or other conventional light microscopy techniques for structural studies of MVEs. In this work, we took advantage of recent developments in super-resolution microscopy to probe the structure of MVEs. Super-resolution microscopy allows 3D imaging of specific cellular structures with spatial resolutions on the 20 nm (lateral) to 40 nm (axial) scales and across an axial range of 1.5–2 µm in a single session. These capabilities make super-resolution microscopy ideally suited for studying the structure of MVEs in their native cellular environment. Using DNA-PAINT, a super-resolution microscopy technique, we were able to reconstruct wholistic views of the MVEs in intact cells with nanometer spatial resolution for the first time. Our results showed that the accumulated vesicles were indeed MVEs characterized by the presence of intraluminal vesicles. The 3D super-resolution images also revealed the rich morphological features of the MVEs and the different spatial arrangements of the ILVs within the enclosing compartment. Although only CD63 was used as an MVE marker for super-resolution imaging, future work will leverage the multiplexed imaging capability of DNA-PAINT and incorporate additional components involved in the exosome secretion process^35,36^. The technology is complementary to the elegant electron tomographic studies of MVEs in the field^41^.

To test whether the exocyst physically directs the trafficking of MVEs, we used an ectopic targeting strategy. We engineered a mitochondrion localized Sec3 through its fusion to Tom20N and then examined whether MVEs would “follow” Sec3 to mitochondria rather than the plasma membrane, their physiological destinations. We found that ectopically localized Sec3 was sufficient to recruit Exo84 to mitochondria, suggesting the assembly of the exocyst complex. Importantly, MVEs were directed to mitochondria, as demonstrated by fluorescence imaging of CD63 and Rab27a, and further verification by electron microscopy. The results from the ectopic targeting assay, together with the biochemistry and imaging analysis, strongly suggest a role of the exocyst in targeting, and probably the physical tethering, of MVEs to the plasma membrane.

Phosphoinositides constitute a defining factor of intracellular membrane compartments, and different phosphoinositide species mediate the recruitment of specific sets of proteins that direct different routes of vesicular trafficking^23–25^. Our study showed that the exocyst associates with the CD63-positive MVEs, and this interaction depends on PI(4)P on these MVEs. A recent study using LC-MS/MS has found that PI(4)P is enriched in the macrophage-derived small EVs^70^. We further identified PI4KIIα as the kinase that catalyzes the production of PI(4)P on MVEs for exocyst recruitment. Interestingly, the exocyst was also reported to co-immunoprecipitate with PI4KIIα^28^. MTM1 is needed to first hydrolyze PI(3)P to PI prior to the generation of PI(4)P. We found that knocking down MTM1 inhibited the association of the exocyst with MVEs. Using an engineered rapamycin-inducible rapid recruitment system in live cells, we demonstrate that the presence of MTM1 on MVEs promoted the recruitment of Exo70. We speculate that the sequential actions of MTM1 and PI4KIIα convert PI(3)P to PI(4)P, which marks MVEs for exocyst-mediated exocytic trafficking and ultimately, exosome secretion. In this regard, the PI switch is probably the upstream “fate determination” step for MVEs in cells. Further supporting this model, we examined PD-L1 trafficking in cells. Inhibition of the exocyst, MTM1, or PI4KIIα by shRNA or chemical inhibitors resulted in PD-L1 re-location to lysosome compartments.

How is the exocyst recruited to PI(4)P positive MVEs? One possibility is that the exocyst binds to PI(4)P. A recent study showed that *Arabidopsis thaliana* Exo70 directly and preferentially binds to PI(4)P, and all the residues necessary for the interaction (K393, K462, K549, K607, and K611) are evolutionarily and functionally conserved in human Exo70^71^. However, it is also possible that the exocyst is recruited to MVEs through binding to Rab proteins, or through both PI(4)P and Rab proteins using a “coincidence detection” mechanism. The Sec15 subunit of the exocyst was reported to directly bind to GTP-Rab11, Rab10 and Rab8^18,22,72^, and these Rab GTPases have been reported to play a role in exosome secretion^13,14,70,73^. Consistent with this notion, we find that both PI(4)P and Rab11 were needed for the localization of the exocyst to the MVEs. This observation, on the other hand, cannot rule out the possibility that PI(4)P recruits the exocyst through the Rab GTPase(s). An early study in yeast showed that PI(4)P recruits Sec2p, the guanine nucleotide exchange factor (GEF) of the exocytic Rab protein Sec4p^74^; it would be interesting to test whether this interaction is conserved in mammalian cells. In addition, MTM1 was reported to interact with GTP-Rab11^29^. Together, MTM1, PI4KIIα, and Rab GTPases may form a module that marks a subpopulation of MVEs for exocyst recruitment and exosome secretion.

In the future, it would be interesting to investigate how MTM1 and PI4KIIα themselves are recruited to the MVEs to initiate the PI switch. Is this a stochastic event or a regulated process controlled by signaling molecules? Does cargo loading play a role in this process? In tumor cells, increased secretion of exosomes and selective enrichment of specific cargo proteins including integrins, EGFR, Jagged-1, or PD-L1^33,73,75–77^ to the exosomes have been observed in response to oncogenic signaling. It would be interesting to investigate whether and how the recruitment of MTM1 and PI4KIIα to MVEs are regulated under different pathophysiological conditions such as cancer.

## Methods

### Plasmids

pEGFP-rExo70 was cloned by inserting rat Exo70 sequence into the pEGFP vector. pLJM1-Flag-rExo70 was cloned by inserting rat Exo70 into the pLJM1-EGFP vector with the GFP sequence removed. Tom20 (1-47aa) was cloned into the pEGFP and pEGFP-Sec3 to create mitochondria-targeting constructs. pEGFP-FYVE, pEGFP-P4C, pEGFP-2xP4M, pEGFP-PI4KIIα, and pEGFP-PI4KIIα-D308A were gifts from the laboratory of Professor Michael Marks (University of Pennsylvania). PI4KIIα and PI4KIIα-D308A sequences were cloned into p3XFLAG-CMV™-14 expression vector (Sigma) to create Flag-tagged PI4KIIα. pEYFP-C1-PLCδ-PH was a gift from Professor Tobias Meyer (Stanford University). pCT-mScarlet-CD63 was a gift from Dr. Jun Wan (Stanford University). mCherry-FKBP-MTM1 was generated by Dr. Tamas Balla (NIH) and purchased from Addgene (#51614). pCR3.1-RFP-CD63 was a gift from (Prof. Bieniasz Rockefeller University). iRFP-FRB-CD63 was generated by subcloning CD63 into the iRFP-FRB vector (#51613, Rab7 removed).

### Antibodies and reagents

Antibody information is included in Supplementary Table 1. PI-273 (HY-103489) was purchased from MedChemExpress. Endosidine-2 (ES2) was purchased from Sigma (1839524-44-5). from ES2 used for DNA-PAINT was purchased from Cayman Chemical (#21888). Rapamycin (37094) was purchased from Millipore (37094).

### Cell lines and cell culture

MDA-MB-231 cells (Georgetown Lombardi Comprehensive Cancer Center (LCCC)), PANC-1 (ATCC), and NMuMG-sfGFP-Exo70 (Gift from Dr. Ian G. Macara) were cultured in DMEM (Invitrogen) supplemented with 10% (v/v) FBS, 100 U/mL of penicillin, and 100 of μg/mL streptomycin, and incubated at 37 °C with 5% (vol/vol) CO_2_. For DNA-PAINT, COS-7 (ATCC) cells were maintained in Gibco DMEM (Thermo Fisher Scientific, 11-995-073) supplemented with 10% fetal bovine serum (Thermo Fisher Scientific, A3840202), and incubated at 37 °C with 5% (vol/vol) CO_2_.

MDA-MB-231 cell lines with stable knockdown of different genes were generated using shRNAs shown in Supplementary Table 2. A scramble sequence-containing plasmid (Addgene) was used as control shRNA. Plasmids expressing shRNAs were packaged into lentiviral particles using HEK293T cells co-transfected with the viral packaging plasmids (RRE, REV, and VSVG). Lentiviral supernatants were collected 48 h after transfection and cell debris were removed by passing through a 0.45-μm filter. Target cells were infected with filtered lentivirus for 24 h and then selected by 10 μg/mL puromycin. pCT-mScarlet-CD63 was packaged using the protocol mentioned in shRNA plasmids packaging. Plasmids and Lipofectamine 2000 (Thermo Fisher Scientific) were mixed according to the manufacturer’s protocol. Cells were refreshed with Opti-MEM and the mixture was added for 4–6 h. The Opti-MEM was removed and replaced with a new DMEM medium. Cells were collected for either immunoblotting or used for immunofluorescence analysis.

### Real-time quantitative PCR

Total RNA was isolated from cells using Purelink RNA mini kit (Invitrogen), and reverse transcribed into cDNA with PrimeScript™ RT Reagent Kit (Takara). The samples were then analyzed in an Applied Biosystems QuantStudio 3 Real-Time PCR system. GAPDH was used as an internal control.

Primers used for MTM1, GAPDH:

qP-MTM1-F GGCCCCATTAAGGGAAGAGTT

qP-MTM1-R CTTGTCGCGCCTCCCATTT

qP-GAPDH-F CAACGGATTTGGTCGTATTG

qP-GAPDH-R GCAACAATATCCACTTTACCAGAGTTAA

### Isolation of small extracellular vesicles (sEV)

For the collection of sEVs, equal numbers of cells were washed with PBS 2 times and cultured in the exosome-free DMEM. After 24 h (sEV secretion level comparison) or 48 h (characterization of exosomes, cargo trafficking), supernatants of the culture were centrifuged at 3000 × *g* for 20 min (Beckman Coulter, Allegra X-14R) to remove cell debris. Microvesicles were pelleted by centrifugation at 16,500 × *g* for 40 min (Beckman Coulter, J2-HS). Supernatants from the above step were then centrifuged at 120,000 × *g* for 16 h at 4 °C (Beckman Coulter, Optima XPN-100, Type 45Ti rotor/Type SW32 Ti rotor). The pellet was suspended in an equal volume of PBS and collected by ultracentrifugation at 120,000 × *g* for 4 h. Then, 100 μL PBS or lysis buffer was first used to resuspend the final sEV pellet. The resuspended sEVs were dissolved completely by pipetting and vortexing, and the final concentration was less than 1.5 μg/μL, which allowed us to reach a relatively homogenous solution without pellets or sticky lipid fractions. More solvent was added and the final volume of the sample was equalized if the sample was not homogenous. For western blotting, 200 μM ES2 or 2 μM PI-273 was used for cell treatment and sEV collection.

### Characterization of isolated exosomes

For morphology verification of isolated exosomes using negative staining electron microscopy, Pellets resuspended in PBS were placed on formvar/carbon-coated nickel grids. After staining with 2% uranyl acetate, grids were air-dried and visualized using a JEM-1011 transmission electron microscope (JEOL USA).

The size and concentration of exosomes isolated from cell culture supernatants were determined using a NanoSight NS300 (Malvern Instruments).

### Transmission electron microscopy

Cells were fixed with a mixture of 2.5% glutaraldehyde, and 2.0% paraformaldehyde in 0.1 M sodium cacodylate buffer (pH 7.4) for 15 min and kept at 4 °C overnight. After 2 times PBS wash, the samples were post-fixed in 2.0% osmium tetroxide with 1.5% K_3_Fe(CN)_6_ for 1 h at room temperature and rinsed in ddH_2_O. After dehydration through a graded ethanol series, the sample was infiltrated and embedded in EMbed-812 (Electron Microscopy Sciences, Fort Washington, PA). Thin sections were stained with uranyl acetate and SATO lead and examined with a JEM1011 electron microscope.

### Immunofluorescence staining and live-cell imaging

For immunofluorescence staining, cells were washed once with PBS and fixed in 4% PFA in PBS for 15 min. The fixed cells were washed 3 times with PBS and permeabilized with 0.1% Triton X-100 before blocking with bovine serum albumin (BSA) for 1 h. The fixed cells were incubated with primary antibodies with recommended dilution overnight at 4 °C, followed by incubation with fluorophore-conjugated secondary antibodies for 2 h. Secondary antibody that targets the IgG1 subtype was used for Exo84 staining. Nuclei were stained with DAPI. For drug treatment, ES2 was diluted in Leibovitz’s L-15 medium at a final concentration of 100 μM. Cells were treated for 4 h before imaging. Washout experiments were performed after 4 h of treatment with ES2, and cells were kept in a fresh medium for 2 h before fixation. Images were acquired using a Nikon confocal microscope at 100x magnification. For each experiment, all images were taken under the same confocal settings. The following setting includes the range of parameters used: laser/filters (405 nm for DAPI, 488 nm for GFP, 515 nm for YFP, 561 nm for mScarlet, 640 nm for Infar-red/iRFP); laser power (10–60%, depending on the specific experiment), exposure time (50–800 ms), digital gain (multiplier 150–250, EM gain 10x), LUTs were applied with auto to acquire the best signal-to-noise ratio.

For live-cell imaging, cells were washed with PBS and cultured in Leibovitz’s L-15 medium (1x) (Gibco) in 35 mm glass-bottomed (14 mm microwell) dishes (MatTek). Each dish was then placed in the thermostable 37 °C chamber for imaging. All further annotations and management were performed in the Fiji software^78^. For the experiments with recruitment of mCherry-MTM1-FKBP, 1 μM rapamycin was used. For tracking of MVEs in live cells, low laser power (20–40% of the maximum), short exposures (100–300 ms) and a digital gain (Multiplier 150–250, EM Gain 10x) were used to minimize photobleaching. Time-lapse sequences were acquired at 1 s per frame. For each experiment, all images were taken under the same confocal settings.

Fluorescence microscopy was performed using an Eclipse TE2000-U inverted microscope (Nikon) equipped with a PLAN APO x100 1.3 NA objective and Cascade 512B CCD camera (Photometrics) driven by Metamorph imaging software (Molecular Devices). Spinning disc confocal microscopy was performed using an Eclipse TiE inverted microscope (Nikon) equipped with a CSUX1 spinning disc (Yokogawa Electric Corporation).

TIRF microscopy was performed using a CFI60 Apochromat TIRF ×100 1.49 NA oil objective, an iXon3 *DU*-*897* back-illuminated EMCCD camera and a perfect focus system driven by NIS-Elements Advanced Research software (Nikon; version 4.50). The corrected TIRF angle was set to a position slightly above where the fluorescence intensity decreased sharply (TIRF angle 2215, 0–7000 range). Time-lapse sequences were acquired at rates of 1 s and 5 s per frame to capture short- and long-time events, respectively. Low laser power (10% to 20% of the maximum), short exposures (10 ms to 50 ms) and a digital gain (Multiplier 150 to 200, EM Gain 10x) were used to minimize photobleaching.

### Vesicle segmentation and quantification

For vesicle segmentation and quantification, confocal Z-stacks were acquired to reveal all vesicles. After the images were imported to ImageJ. The image intensity was adjusted using the same settings, and the images were converted to 8-bit files. For vesicle segmentation, the LoG detector of TrackMate2 plugin in ImageJ was used with the following settings: the estimated object diameter was set to 10 pixels (~1 μm) for CD63-labeled vesicles and to 6 pixels (~0.6 μm) for EEA1-labeled structures; the quality threshold was set to 4 for CD63 and 12 for EEA1. All other settings were kept as the plugin default. To determine the number of vesicles, the preview was used to check for the segmentation and to count the numbers. If two vesicles were too close to each other, but not separated by the software, they were manually counted as 2.

For the quantification of exocyst-positive MVEs, all vesicles were first segmented using Trackmate2 as described above. The x and y coordinates of each vesicle were recorded and used to locate vesicles in the original images opened in the NIS-Elements Advanced Research software (Nikon; version 4.50). Then, a 40-pixel-long line was drawn across each vesicle. These lines were used to generate the fluorescence intensity profiles from the original images that were converted to 8-bit files. A peak with intensity above the cutoff of 100 units was defined as a “signal”. If the area of overlap between the vesicle peak (CD63 or EEA1) and exocyst peak (Exo84 or Sec15) was greater than 50% of the exocyst peak area, the vesicle was counted as exocyst-positive. The number of exocyst-positive vesicles *versus* the total MVE number was defined as the percentage of exocyst-positive MVEs.

The same procedure was used to quantify the phosphoinositide intensity on MVEs labeled by CD63.

### Quantification of MVE-mitochondria contact sites (MMCS)

For MMCS quantification, we adapted the procedure previously described for quantification of the ER-endosome membrane contact sites^49,79^. Specifically, all CD63 vesicles were segmented using the above method using the Trackmate2 function except that 50-pixel long lines were used for intensity profiles. These lines were drawn across each individual vesicle toward the closest mitochondrion. If there was an overlap between the signals from the two channels in the resulting intensity profiles, they were considered as MMCSs.

For quantification of MMCS in different areas of the cells, the perinuclear region was defined as having a diameter twice the nuclear diameter of the selected cell. The remaining cell area was defined as the peripheral region. For EM images, the distance between MVEs and mitochondria that was less than the average radius of MVEs was considered as a direct contact.

### 3D rendering of confocal immunofluorescence images

Confocal images with Z-stacks information were processed to 3D modeling by NIS-Elements Advanced Research software (Nikon; version 4.50). Briefly, the 3D reconstruction of confocal stacks was generated from 8 to 10 consecutive z slices with a thickness of 0.2–0.3 µm using the alpha blending option in the NIS-Elements software.

### Cell culture and immunostaining for DNA-PAINT

COS-7 cells were plated in a Lab-Tek® II 8-well chambered cover glass (ThermoFisher, 155360) for 24 to 48 h until reaching the desired confluence (~70–80%). Cells were treated with 100 µM ES-2 (Cayman Chemical, #21888, 50 mM stock solution was prepared in DMSO) for 4 h prior to fixation. Control cells were treated with equal volumes of DMSO. Cell fixation and immunostaining were processed as described before^36^. Briefly, cells were fixed with 3.7% paraformaldehyde (PFA) in 1x PHEM buffer at room temperature for 20 min. After three DPBS (ThermoFisher, 14-190-144) washes, cells were quenched and permeabilized with 300 mM glycine (Sigma-Aldrich, 50046) in DPBS containing 0.3% saponin (Sigma-Aldrich, 47036) for 30 min. Cells were then rinsed with DPBS and blocked with Image-iT® FX signal enhancer (ThermoFisher, I36933) at room temperature for 30 min. Following three washes with DPBS, cells were blocked with 3% bovine serum albumin (Fisher Scientific, BP1600) and 5% salmon sperm DNA (ThermoFisher, AM9680) in DPBS for 30 min on a rocker. Cells were then incubated with a mouse monoclonal anti-CD63 antibody (Abcam, ab8219, dilution ratio 1:250) in the same BSA blocking buffer at room temperature on a rocker for 90 min. Cells were then rinsed in DPBS three times, 5 min each. Next, cells were incubated with DNA-conjugated donkey anti-mouse IgG (H+L) (Jackson Immuno Research, 715-005-150, dilution ratio 1:200) in DPBS with 1% BSA and 5% salmon sperm DNA at room temperature for 1 h. Here, donkey anti-mouse secondary antibodies were pre-conjugated with DNA oligos, namely DS1 (sequence: 5’-TATACATCTAAATACATCTAAT), using a procedure as previously described^36^. Cells were rinsed three times with DPBS. Next, cells were post-fixed with 3.7% PFA and 0.1% glutaraldehyde in a PHEM buffer at room temperature for 10 min, which is critical to subsequent DNA-PAINT imaging. Finally, cells were washed in DPBS three times. Before DNA-PAINT imaging, gold nanoparticles (BBI Solutions, EM.GC50) were added to samples for fiducial-based drift correction and image registration.

### 3D DNA-PAINT imaging

All 3D DNA-PAINT super-resolution fluorescence data were taken on a custom-built single-molecule imaging system used in our previous work^80^. Additionally, a cylindrical lens (f = 1000 mm, Thorlabs, LJ1516L1-A) was used to introduce astigmatism in the detection path. This method allows the extraction of z position from the shape of the astigmatic point-spread function (PSF). All imaging was performed using Atto643 (Atto-tec, AD643) labeled imager strands IS1 (sequence: 5’-TTAGATGTAT) in imaging buffer C (1x PBS with 500 mM NaCl) containing 12% ethylene carbonate (EC) (Sigma-Aldrich, 676802). For each imaging dataset, typically 50,000 frames of raw DNA-PAINT imaging were acquired at 50 ms exposure time using micromanager^81^.

### 3D image data analysis and rendering

Image analysis, including single-molecule localization and subsequent coordinate filtering, sorting, and rendering was performed using an open-source single-molecule localization microscopy (SMLM) software platform, SMAP^82^. To be able to extract z-positions of the localizations, a z-calibration dataset was collected. By this mean, z-stacks of 10–20 fields of view of TetraSpeck beads were acquired with a 20 nm step size. This beads imaging was used to generate a model of the experimental point-spread function (PSF) that was subsequently employed to fit single-molecule localizations. The assorted localizations were then used for final image rendering with color-coded position in the z-axis, and the rendered images were exported as TIF files for further analysis and annotations in Fiji^78^.

Exported x, y, and z coordinates of filtered localizations from SMAP were scaled by a factor of 15 and saved in.XYZ file format with all localizations given matching pseudoatoms of Uranium.

Utilizing UCSF Chimera software^39^ version 1.14,.XYZ files were loaded with the resulting rendered scale of 13.46 nm (real distance from SMAP) per 1 Å (pseudo rendered distance in Chimera). These localization clouds were converted into density volume approximations with the built-in “molmap” function using a resolution of 1.4 and a sigma factor of 1.5–2 depending on the localization density from SMAP filtering, resulting in a final rendered resolution of 24–32 nm. Spurious localizations were filtered using the Hide Dust volume function at 1.5 voxels, removing individual localizations ~60 nm or more from other structures. Potential internal structures were pseudo-colored using Color Zone with a voxel radius of 8–10 depending on overall vesicle localization density. Cross-section views were generated using the Side View function with depth perspective indicated by diverging lines and a white outer bounding box indicating the exported region from SMAP.

### Western blotting

Protein samples were separated using 12% SDS–PAGE and transferred onto nitrocellulose membranes. The blots were blocked with 5% non-fat dry milk in TBST at room temperature for 1 h and incubated overnight at 4 °C with primary antibodies at dilutions recommended by the suppliers, followed by incubation with HRP-conjugated secondary antibodies (Cell Signaling Technology). The blots on the membranes were developed with ECL detection reagents (Pierce) or Chemiluminescent HRP substrates (Millipore WBKLS0500) under KuikQuant Imager (Kindle Biosciences). Densitometry for western blotting was quantified by ImageJ (Fiji) as recommended by Fiji’s website (30.13–Gels). Briefly, bands were converted into 8-bit images, selected by a rectangular selection, and plotted as suggested. Areas were measured by the wand tool and normalized for the final statistics.

### Statistical analyses

Statistical analyses were performed using GraphPad Prism v.8.3. For normally distributed data, the significance of mean differences was determined using two-sided unpaired Student’s *t*-test, one-sample *t*-test was used to the knockdown efficiency of MTM1 shRNAs. Pearson’s correlation coefficient was derived from NIS-Elements Advanced Research software (Nikon; version 4.50) using raw uncropped whole images of cells. Error bars shown in graphical data represent mean ± s.d. A two-tailed value of *P* < 0.05 was considered statistically significant.

### Reporting summary

Further information on research design is available in the Nature Portfolio Reporting Summary linked to this article.

### Supplementary information

Supplementary Information

Description of Additional Supplementary Files

Supplementary Movie 1

Supplementary Movie 2

Supplementary Movie 3

Supplementary Movie 4

Supplementary Movie 5

Reporting Summary

### Source data

Source Data

## Data Availability

All data supporting the findings of this study are available from the corresponding author on request. Source data are provided with this paper.
