# Supplementary material for: A phosphoinositide switch mediates exocyst recruitment to multivesicular endosomes for exosome secretion
Source: Nat Commun. 2023 Oct 28;14:6883. doi: 10.1038/s41467-023-42661-0 (PMC10613218; doi:10.1038/s41467-023-42661-0)
Supplement: Supplementary file 1 — Supplementary Information [file 41467_2023_42661_MOESM1_ESM.pdf]

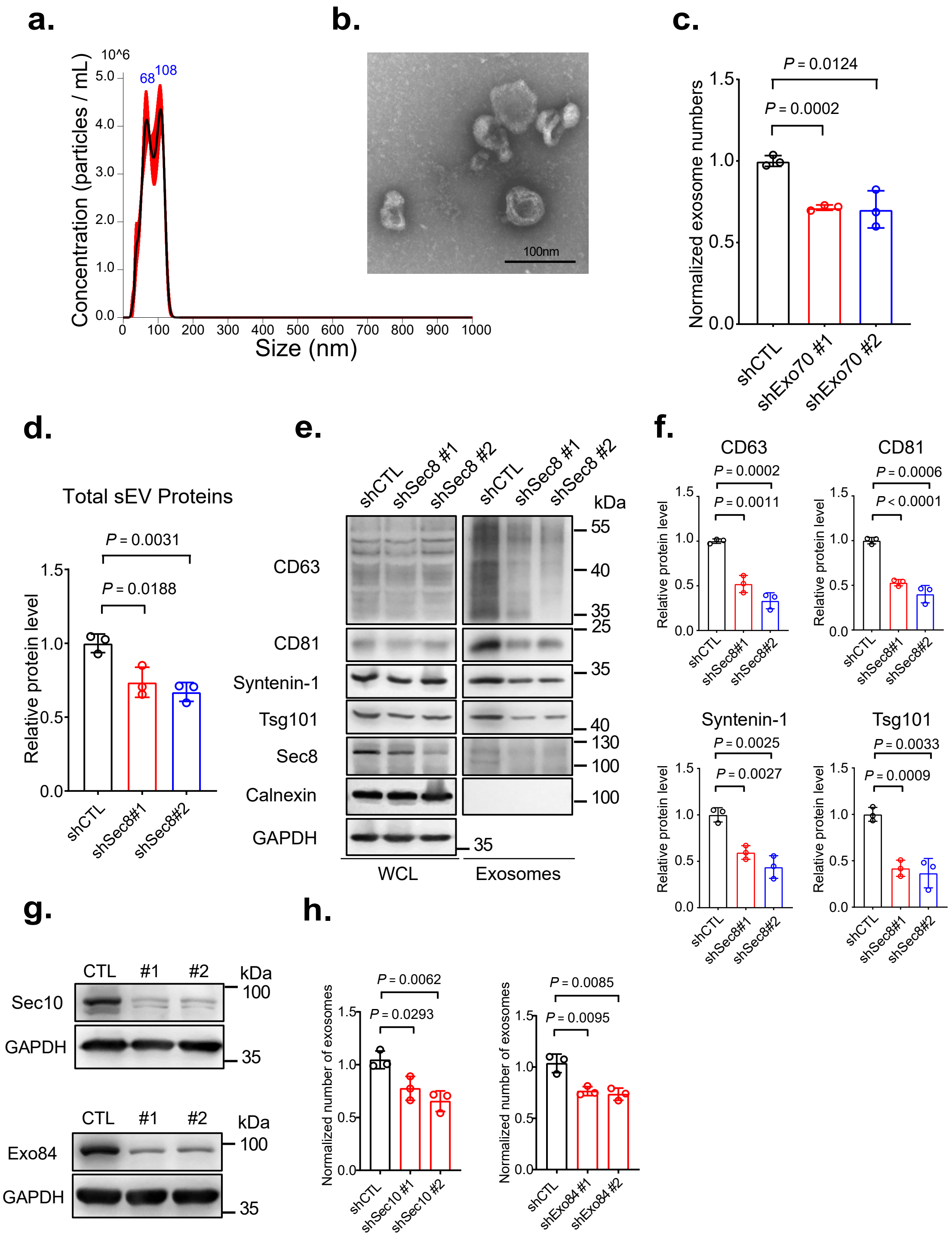
a.
10^6
68
108
5.0
4.0
3.0
2.0
1.0
0.0
Concentration (particles / mL)
0
100
200
300
400
500
600
700
800
900
1000
Size (nm)
b.
100nm
c.
P = 0.0124
P = 0.0002
Normalized exosome numbers
shCTL
shExo70 #1
shExo70 #2
d.
Total sEV Proteins
P = 0.0031
P = 0.0188
Relative protein level
shCTL
shSec8#1
shSec8#2
e.
shCTL
shSec8 #1
shSec8 #2
kDa
CD63
CD81
Syntenin-1
Tsg101
Sec8
Calnexin
GAPDH
WCL
Exosomes
f.
CD63
P = 0.0002
P = 0.0011
CD81
P = 0.0006
P < 0.0001
Syntenin-1
P = 0.0025
P = 0.0027
Tsg101
P = 0.0033
P = 0.0009
Relative protein level
g.
CTL
#1
#2
Sec10
GAPDH
Exo84
h.
P = 0.0062
P = 0.0293
Normalized number of exosomes
shCTL
shSec10 #1
shSec10 #2
P = 0.0085
P = 0.0095
shExo84 #1
shExo84 #2

Supplementary Data Fig. 1. Exo70 and Sec8 regulate the secretion of exosomes. a. A representative NTA of size distribution of exosomes isolated from MDA-MB-231 cell media. Blue numbers represent the peak size of EVs. Red area represents the variance. **b.** A representative negatively stained TEM image of isolated exosomes from MDA-MB-231 cells. Scale bar = 100 nm. **c.** NTA of exosome secreted from cells stably expressing control or Exo70 shRNAs (n = 3). The average number of exosomes isolated from cells expressing Exo70 shRNA is normalized to that of control samples. **d.** Quantification of exosomal proteins from cells treated with control and Sec8 shRNAs (n = 3). **e.** Western blot analysis of whole cell lysates (WCL) and exosomes isolated from cells stably expressing either control or Sec8 shRNAs. Exosomes from equal amounts of cells ($1x10^7$) were collected for Western blot analysis. **f.** Quantification of the levels of CD63, CD81, Syntenin-1, and Tsg101 on exosomes from the cells expressing either control or Sec8 shRNAs (n = 3). The average amount of exosomal proteins from cells expressing Sec8 shRNA is normalized to that from control samples. **g.** Western blot analysis of shRNA knockdown of Sec10 and Exo84. **h.** NTA of exosome secretion from cells stably expressing either control, Sec8, or Exo84 shRNAs (n = 3). The average number of exosomes isolated from cells expressing Sec8 or Exo84 shRNA is normalized to that of control samples. Data are presented as mean ± s.d. of three independent biological replicates. P values are calculated using two-sided unpaired t-test.

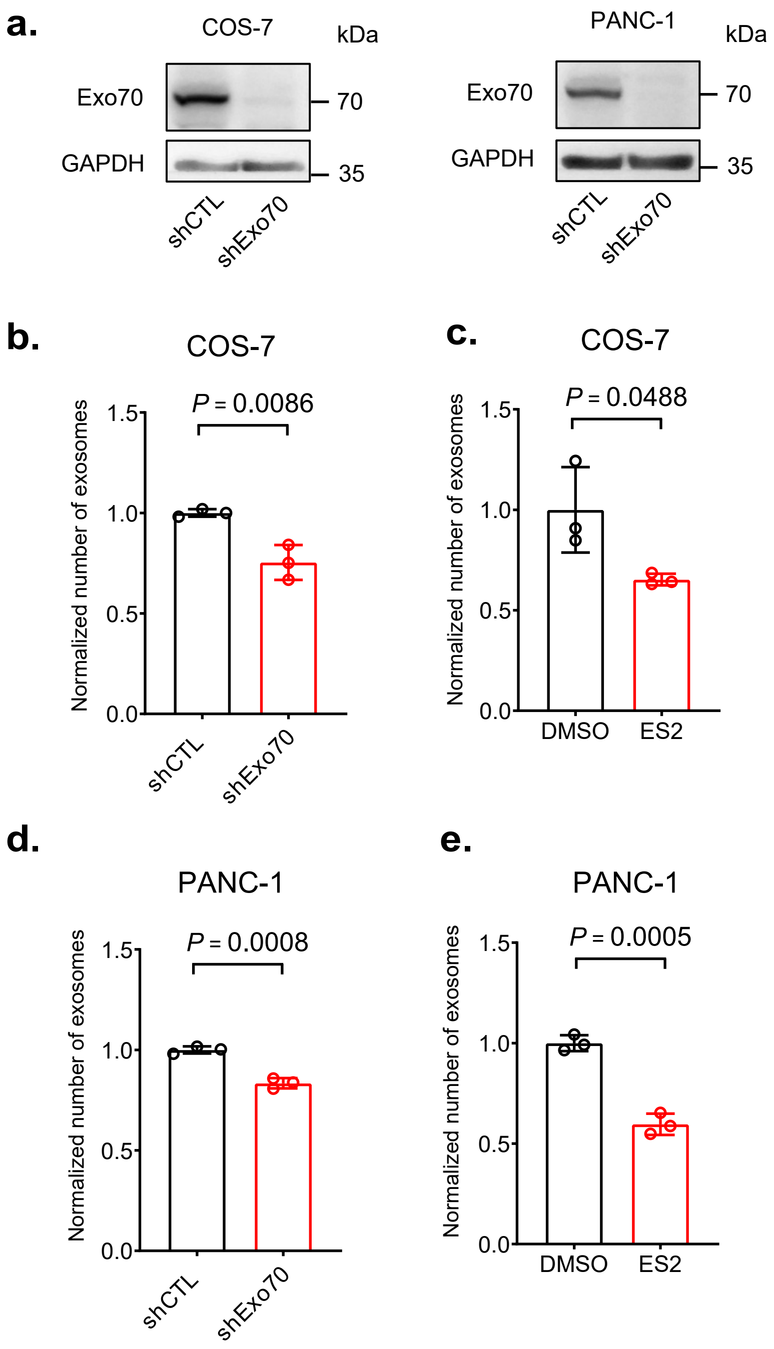
a.
COS-7
kDa
Exo70
70
GAPDH
35
shCTL
shExo70
PANC-1
kDa
Exo70
70
GAPDH
35
shCTL
shExo70
b.
COS-7
P = 0.0086
Normalized number of exosomes
1.5
1.0
0.5
0.0
shCTL
shExo70
c.
COS-7
P = 0.0488
Normalized number of exosomes
1.5
1.0
0.5
0.0
DMSO
ES2
d.
PANC-1
P = 0.0008
Normalized number of exosomes
1.5
1.0
0.5
0.0
shCTL
shExo70
e.
PANC-1
P = 0.0005
Normalized number of exosomes
1.5
1.0
0.5
0.0
DMSO
ES2

Supplementary Data Fig. 2. Exo70 regulates the secretion of exosomes in COS-7 and PANC-1 cells. a. Western blotting shows the knockdown of Exo70 in COS-7 and PANC-1 cells expressing shRNA targeting Exo70 as compared with cells expressing scrambled shRNA (shCTL). **b, c.** NTA of exosome secretion from COS-7 cells stably expressing control or Exo70 shRNAs (**b**, n=3) or treated with DMSO or ES2 (**c**, n = 3). The average numbers of exosomes isolated from treated cells are normalized to that in control samples. **d, e.** NTA of exosome secretion from PANC-1 cells stably expressing control or Exo70 shRNAs (**d,** n = 3) or treated with DMSO or ES2 (**e**, n = 3). The average numbers of exosomes isolated from treated cells are normalized that in control samples. Data are presented as mean ± s.d. of three independent biological replicates. P values are calculated using two-sided unpaired t-test.

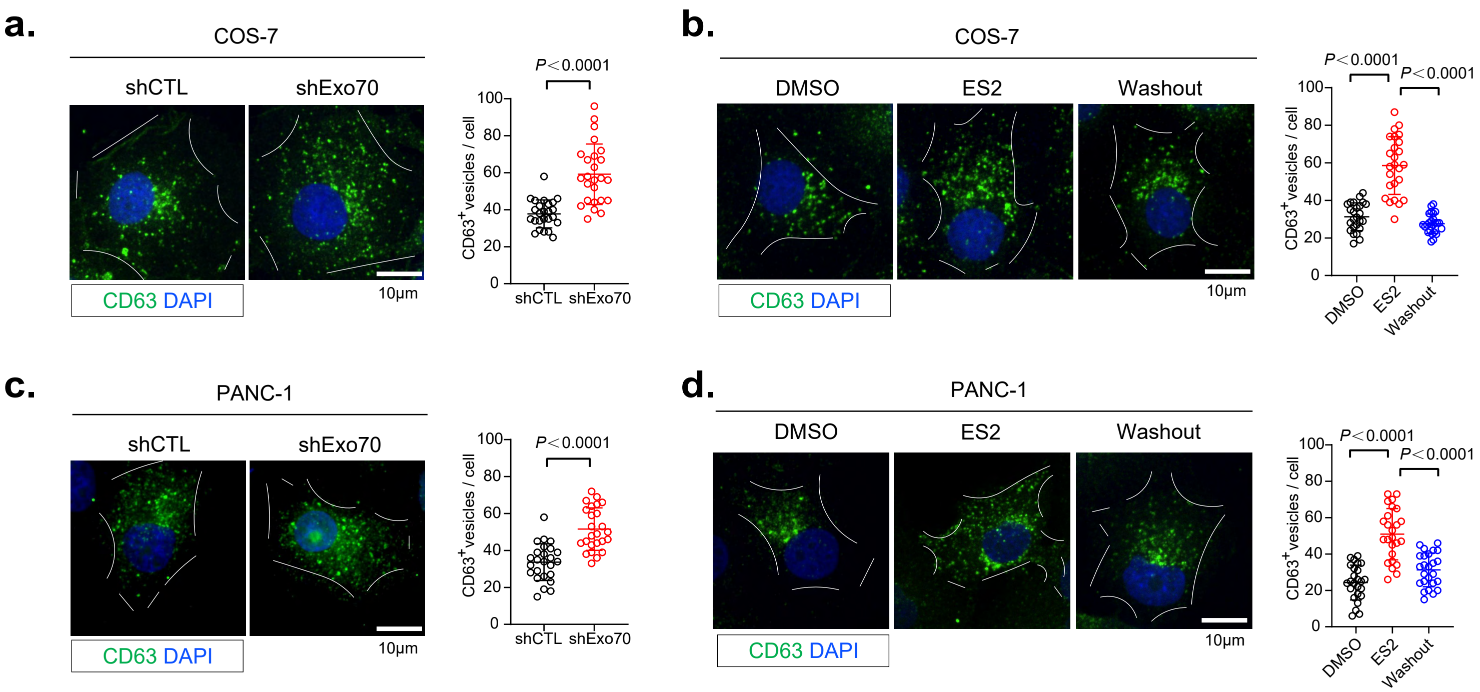
a.
COS-7
shCTL
shExo70
CD63 DAPI
10μm
P<0.0001
CD63+ vesicles / cell
shCTL
shExo70
b.
COS-7
DMSO
ES2
Washout
CD63 DAPI
10μm
P<0.0001
P<0.0001
CD63+ vesicles / cell
DMSO
ES2
Washout
c.
PANC-1
shCTL
shExo70
CD63 DAPI
10μm
P<0.0001
CD63+ vesicles / cell
shCTL
shExo70
d.
PANC-1
DMSO
ES2
Washout
CD63 DAPI
10μm
P<0.0001
P<0.0001
CD63+ vesicles / cell
DMSO
ES2
Washout

Supplementary Data Fig. 3. Exo70 regulates the trafficking of MVEs in COS-7 and PANC-1 cells. a. Imaging (left) and quantification (right) of MVEs marked by CD63 in COS-7 cells stably expressing control or Exo70 shRNAs (n = 25 cells). Scale bar = 10 μm. **b.** Imaging (left) and quantification (right) of MVEs marked by CD63 in COS-7 cells treated with DMSO, ES2, or after washout of ES2. Scale bar = 10 μm (n = 25 cells). **c.** Imaging (left) and quantification (right) of MVEs marked by CD63 in PANC-1 cells stably expressing the control or Exo70 shRNAs (#1 and #2) (n = 25 cells). **d.** Imaging (left) and quantification (right) of MVEs marked by CD63 in PANC-1 cells treated with DMSO, ES2, or after washout of ES2. Scale bar = 10 μm (n = 25 cells). P values are calculated using two-sided unpaired t-test.

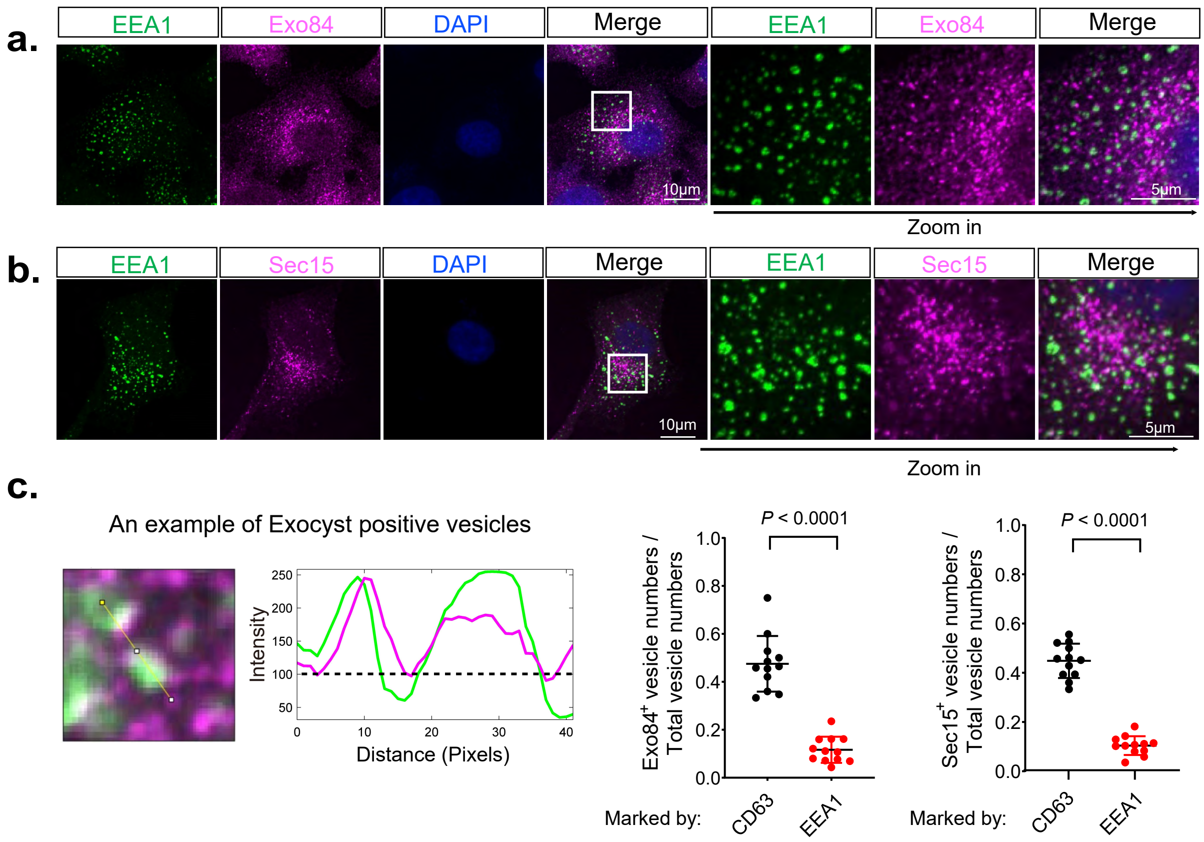

a.
EEA1
Exo84
DAPI
Merge
10μm
EEA1
Exo84
Merge
5μm
Zoom in
b.
EEA1
Sec15
DAPI
Merge
10μm
EEA1
Sec15
Merge
5μm
Zoom in
c.
An example of Exocyst positive vesicles
Intensity
Distance (Pixels)
P < 0.0001
Exo84+ vesicle numbers / Total vesicle numbers
Marked by:
CD63
EEA1
P < 0.0001
Sec15+ vesicle numbers / Total vesicle numbers
Marked by:
CD63
EEA1

Supplementary Data Fig. 4. Analysis of the association of Exo84 and Sec15 with vesicles. a. Immunostaining of Exo84 and EEA1. Zoom-in view of the boxed region is shown at the right. **b.** Immunostaining of Sec15 and EEA1. Zoom-in view of the boxed region is shown at the right. Scale bars are indicated in the panels. **c.** A representative image of Exo84 association with CD63 vesicles is shown at the left. The intensity profile shows two vesicles with colocalized Exo84-CD63 signal above the background signal. Analysis of exocyst positive subpopulation of MVEs (CD63) or early endosomes (EEA1) (see *Materials and Methods*) are shown at right (n = 12 cells). P values were calculated using two-sided unpaired t-test.

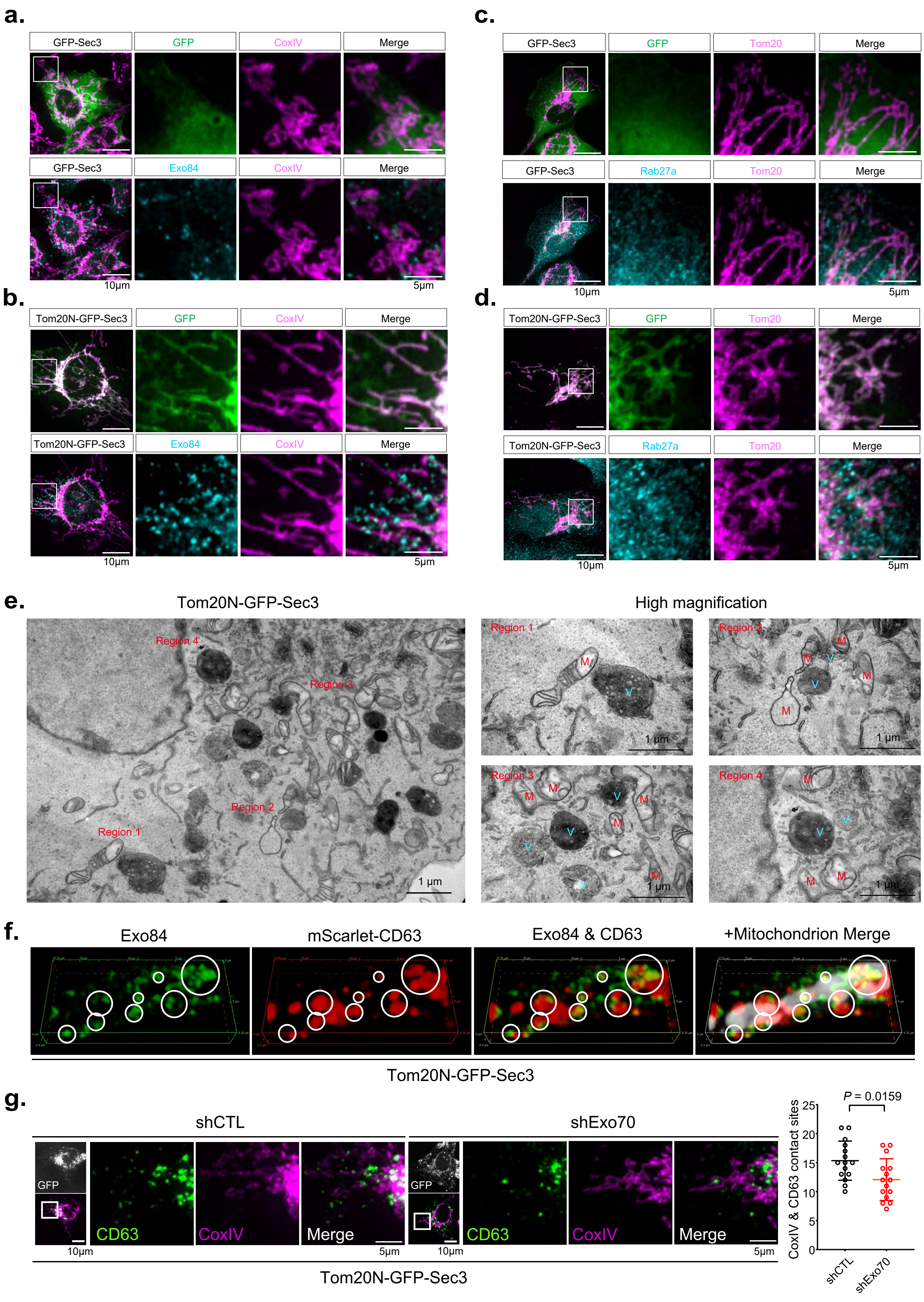
a.
GFP-Sec3
GFP
CoxIV
Merge
Exo84
10μm
5μm
b.
Tom20N-GFP-Sec3
c.
Tom20
Rab27a
d.
e.
Tom20N-GFP-Sec3
High magnification
Region 1
Region 2
Region 3
Region 4
M
V
1 μm
f.
Exo84
mScarlet-CD63
Exo84 & CD63
+Mitochondrion Merge
g.
shCTL
shExo70
CD63
P = 0.0159
CoxIV & CD63 contact sites
0
5
10
15
20
25

Supplementary Data Fig. 5. Mitochondria-targeted Sec3 redirects Exo84 and Rab27a-positive vesicles to mitochondria. a. Localization of CoxIV, GFP-Sec3, and Exo84 in cells. The boxed regions are magnified on the right. **b.** Localization of CoxIV, Tom20N-GFP-Sec3, and Exo84 in cells. Exo84 is recruited to the mitochondria marked by Tom20N-GFP-Sec3 and CoxIV. **c.** Localization of Tom20, GFP-Sec3, Rab27a in cells. Endogenous Tom20 was stained with an antibody that recognizes the full-length protein but not the Tom20 N-terminal fragment (a.a. 1-47). **d.** Localization of Tom20, Tom20N-GFP-Sec3, Rab27a in cells. Rab27a is recruited to the mitochondria marked by Tom20N-GFP-Sec3. The boxed regions in **c** and **d** are magnified on the right. Scale bars are indicated in the panels. **e.** EM images of a representative cell expressing Tom20N-GFP-Sec3. Four regions are shown at higher magnification. “V”, vesicles, “M”, mitochondria. Direct contacts of mitochondria and MVEs are shown. Scale bars = 1 μm. **f.** 3D reconstruction of the association of MVEs with mitochondria in Tom20N-GFP-Sec3 expressing cells. Regions of colocalization of Exo84 (green) and mScarlet-CD63 (magenta) are indicated by circles. Mitochondria marked by Tom20N-GFP-Sec3 are rendered grey. **g.** Left, Tom20-GFP-Sec3 localized to the mitochondria cells expressing the scramble shRNA and the shRNA targeting Exo70. The quantification of MMCSs was shown on the right (n = 15). Right, Tom20-GFP-Sec3 localized to the mitochondria cells treated with DMSO and ES2 (n = 15). The quantification of MMCSs was shown on the right (n = 15). Scale Bars are indicated in the panels. P values were calculated using two-sided unpaired t-test.

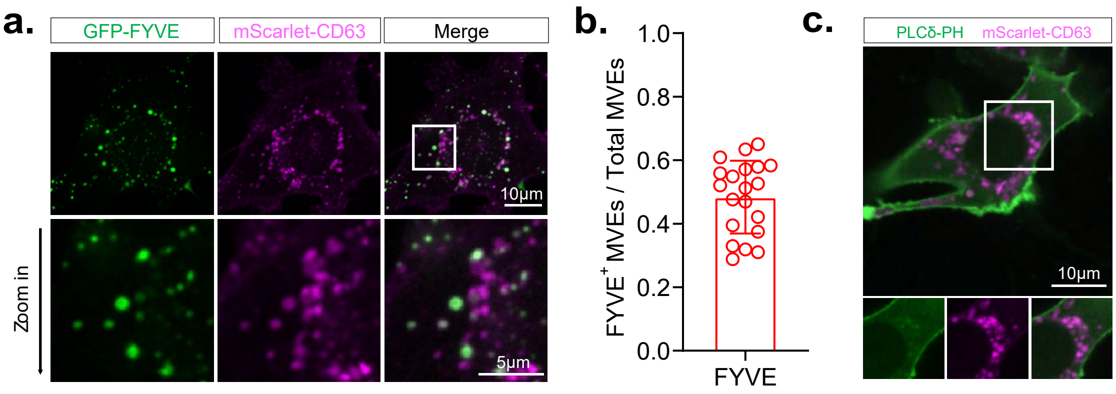
a.
GFP-FYVE
mScarlet-CD63
Merge
10μm
Zoom in
5μm
b.
FYVE^{+} MVEs / Total MVEs
1.0
0.8
0.6
0.4
0.2
0.0
FYVE
c.
PLCδ-PH
mScarlet-CD63
10μm

Supplementary Data Fig. 6. Detection of PI(3)P and PI(4,5)P2 in cells. a. PI(3)P labeled by GFP-FYVE was localized to some of the mScarlet-CD63 positive MVEs. Zoom-in images of the boxed region are shown at the lower panel. **b.** Quantification of the GFP-FYVE positive subpopulation is shown at the right (n = 20 cells). Scale bars are indicated in the panels. P values were calculated using two-sided unpaired t-test. **c.** PI(4,5)P labeled by PLC-PH showed mostly plasma membrane distribution and limited colocalization with mScarlet-CD63 positive MVEs. The boxed region is shown at the lower panel.

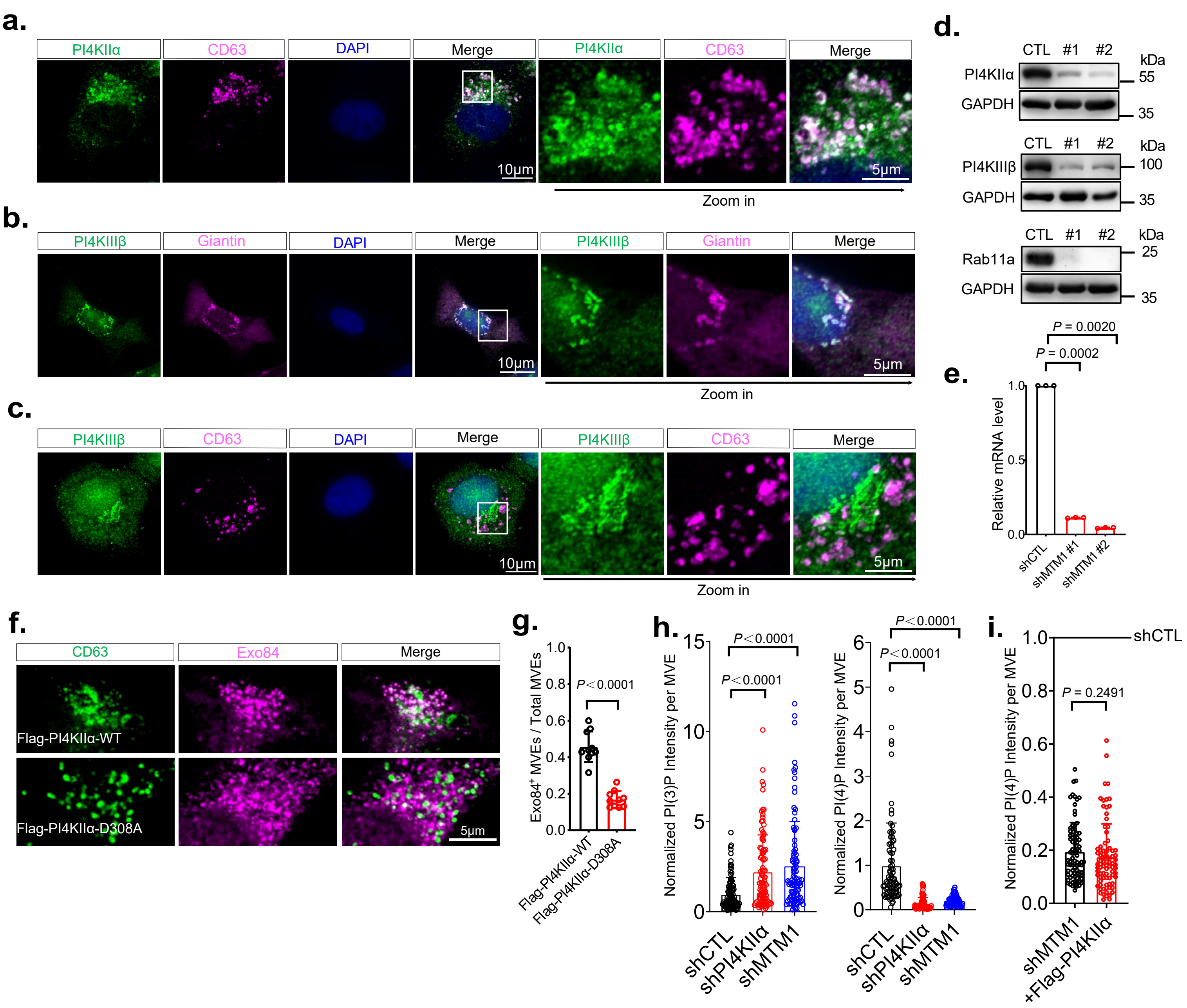
a.
PI4KIIα
CD63
DAPI
Merge
10μm
5μm
Zoom in
b.
PI4KIIIβ
Giantin
DAPI
Merge
10μm
5μm
Zoom in
c.
PI4KIIIβ
CD63
DAPI
Merge
10μm
5μm
Zoom in
d.
CTL #1 #2
kDa
PI4KIIα 55
GAPDH 35
PI4KIIIβ 100
GAPDH 35
Rab11a 25
GAPDH 35
e.
P = 0.0020
P = 0.0002
Relative mRNA level
shCTL
shMTM1 #1
shMTM1 #2
f.
CD63
Exo84
Merge
Flag-PI4KIIα-WT
Flag-PI4KIIα-D308A
5μm
g.
Exo84+ MVEs / Total MVEs
P<0.0001
Flag-PI4KIIα-WT
Flag-PI4KIIα-D308A
h.
Normalized PI(3)P Intensity per MVE
P<0.0001
P<0.0001
shCTL
shPI4KIIα
shMTM1
Normalized PI(4)P Intensity per MVE
P<0.0001
P<0.0001
shCTL
shPI4KIIα
shMTM1
i.
shCTL
Normalized PI(4)P Intensity per MVE
P = 0.2491
shMTM1
+Flag-PI4KIIα

Supplementary Data Fig. 7. PI4KIIα, MTM1, and Rab11 are needed for the recruitment of the exocyst to MVEs. a. Co-localization of PI4KIIα (green) and CD63 (magenta) in cells. Zoom-in images of the boxed region are shown at right. Scale bars are indicated in the panels. **b.** Localization of PI4KIIIβ (green) to the Golgi (marked by Giantin, magenta) in MDA-MB-231 cells. The nucleus is stained with DAPI. **c.** Localization of PI4KIIIβ (green) and CD63 (magenta) in MDA-MB-231 cells. Scale bars are indicated in the panels. **d.** Western blotting showing the shRNA-mediated knockdown of PI4KIIIα, PI4KIIIβ, and Rab11a. **e.** RT-qPCR quantification of MTM1 mRNA level in cells stably expressing control or MTM1 shRNAs. P-values were calculated using one-sample t-tests. **f.** Immunostaining of Exo84 (magenta) and CD63 (green) in stable PI4KIIα knockdown cells (shRNA targeting 3' UTR) expressing Flag-PI4KIIα-WT or Flag-PI4KIIα-D308A. Scale bars = 5 μm. **g.** Subpopulation of Exo84-positive MVEs in stable PI4KIIα knockdown cells expressing Flag-PI4KIIα-WT or Flag-PI4KIIα-D308A (n = 10 cells per condition). P values are calculated using a two-sided unpaired t-test. **h.** Quantification of PI(3)P (n = 99 vesicles) or PI(4)P (n = 83 vesicles) intensity per MVE in cells expressing the control, PI4KIIα, or MTM1 shRNAs. The average phosphoinositide intensity in cells expressing PI4KIIα or MTM1shRNA is normalized to that of control. P values were calculated using two-sided unpaired Welch's t-test. **i.** Quantification of PI(4)P (n = 83 vesicles) intensity level per MVE in MTM1 knockdown cells with or without Flag-PI4KIIα expression normalized to the average PI(4)P intensity level in cells expressing control shRNA (shCTL). P values were calculated using two-sided unpaired t-test unless specifically noted.

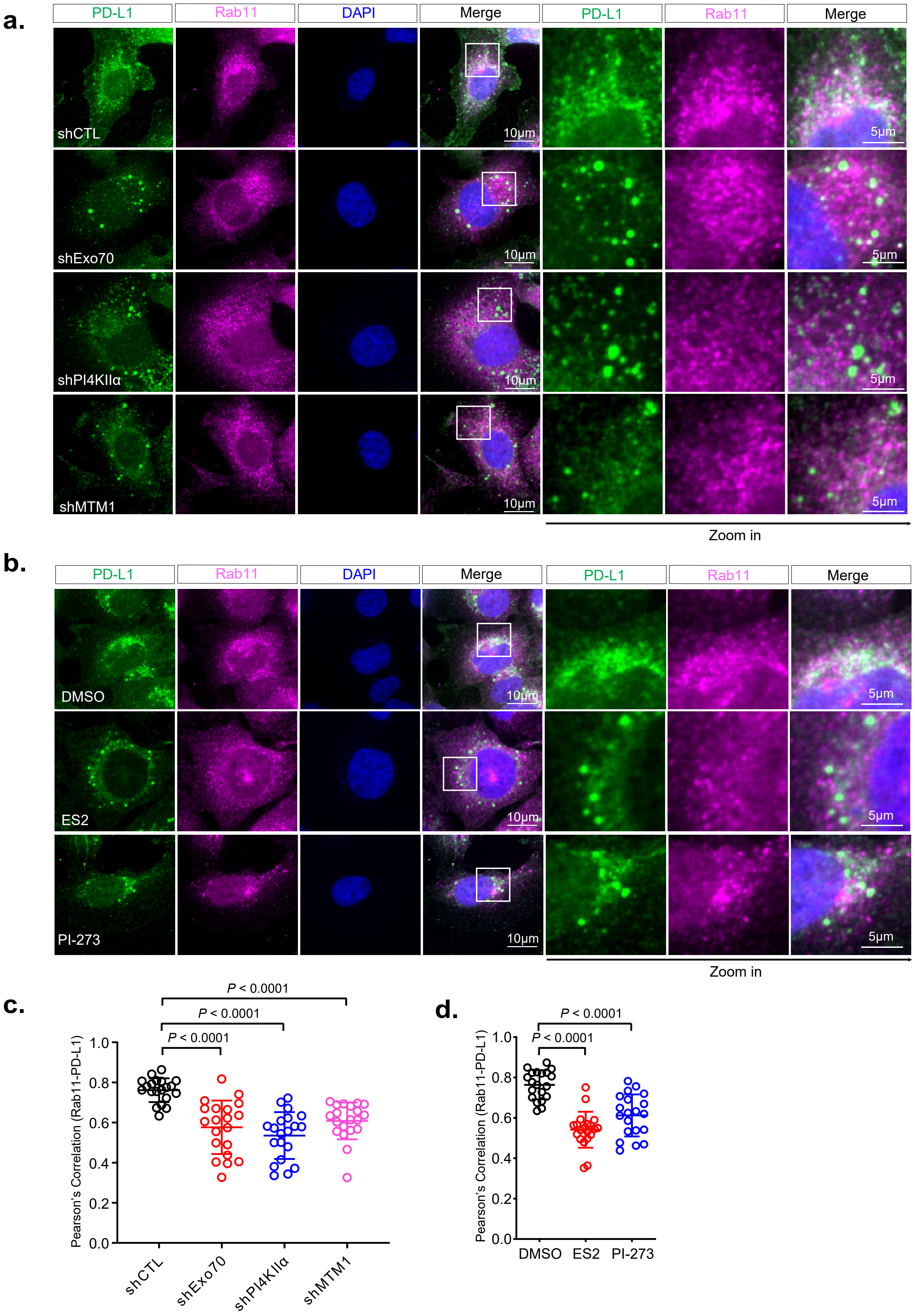
a.
PD-L1
Rab11
DAPI
Merge
PD-L1
Rab11
Merge
shCTL
shExo70
shPI4KIIα
shMTM1
10μm
5μm
Zoom in
b.
PD-L1
Rab11
DAPI
Merge
PD-L1
Rab11
Merge
DMSO
ES2
PI-273
10μm
5μm
Zoom in
c.
P < 0.0001
P < 0.0001
P < 0.0001
Pearson's Correlation (Rab11-PD-L1)
1.0
0.8
0.6
0.4
0.2
0.0
shCTL
shExo70
shPI4KIIα
shMTM1
d.
P < 0.0001
P < 0.0001
Pearson's Correlation (Rab11-PD-L1)
1.0
0.8
0.6
0.4
0.2
0.0
DMSO
ES2
PI-273

Supplementary Data Figure 8. Localization of PD-L1 in cells with inhibition of Exo70, PI4KIIα, or MTM1. a. Localization of PD-L1 and Rab11 in MDA-MB-231 cells stably expressing control shRNA or shRNAs targeting Exo70, PI4KIIα, or MTM1. Zoom-in images of the boxed regions are shown to the right. Scale bar = 10 μm. **b.** Localization of PD-L1 and Rab11 in cells treated with DMSO, ES2, or PI-273. Zoom-in images of the boxed regions are shown to the right. **c.** Colocalization analysis of PD-L1 and Rab11 by Pearson's correlation coefficient in cells treated different shRNAs (n = 20 cells per condition). **d.** Colocalization analysis of PD-L1 and Rab11 by Pearson's correlation coefficient in cells treated with DMSO, ES2, or PI-273 (n = 20 cells per condition). P values are calculated using a two-sided unpaired t-test. Scale bars are indicated in the panels.

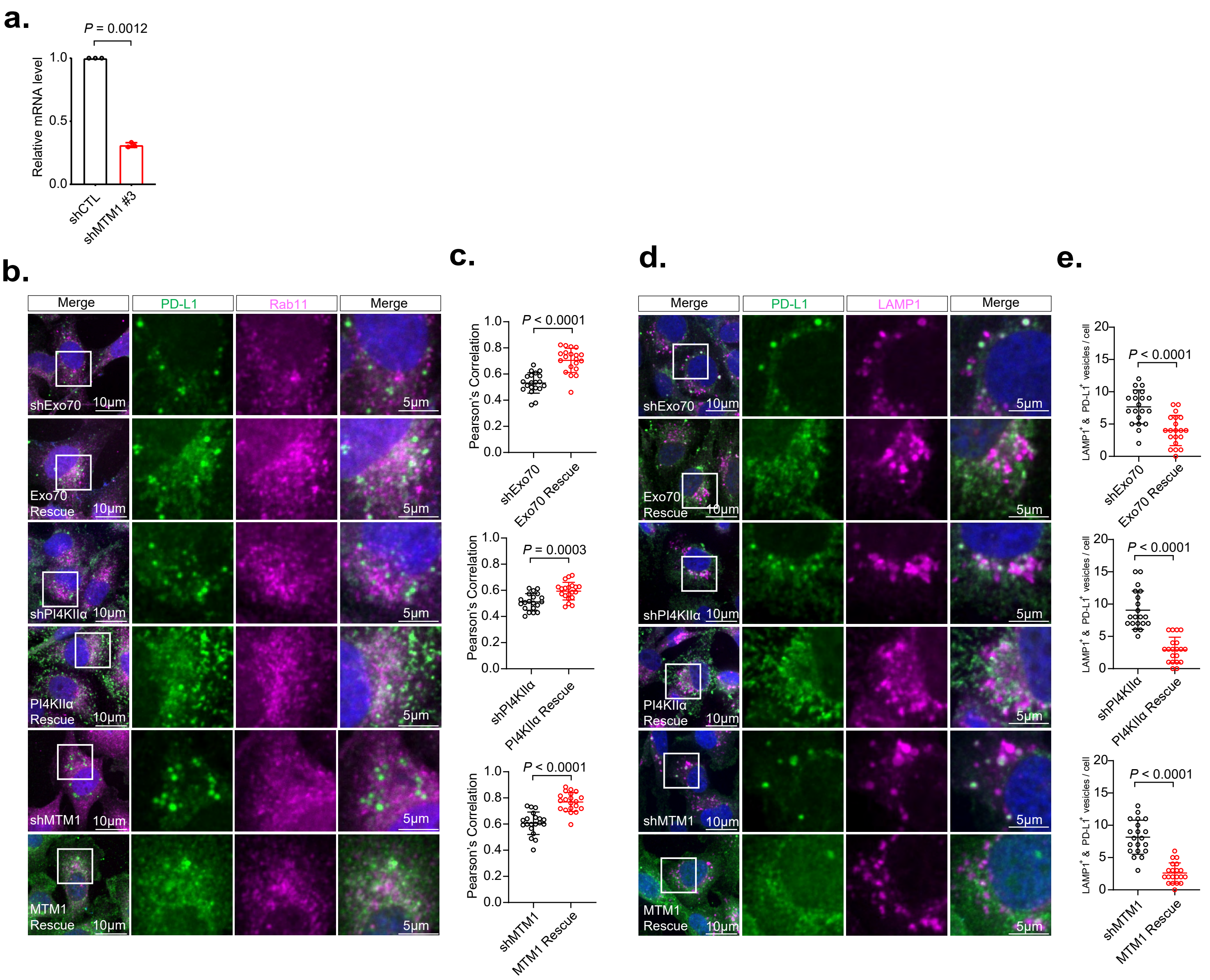

a.
P = 0.0012
Relative mRNA level
1.0
0.5
0.0
shCTL
shMTM1 #3
b.
Merge
PD-L1
Rab11
Merge
shExo70
Exo70 Rescue
shPI4KIIα
PI4KIIα Rescue
shMTM1
MTM1 Rescue
10μm
5μm
c.
Pearson's Correlation
P < 0.0001
P = 0.0003
P < 0.0001
shExo70
Exo70 Rescue
shPI4KIIα
PI4KIIα Rescue
shMTM1
MTM1 Rescue
d.
Merge
PD-L1
LAMP1
Merge
e.
LAMP1+ & PD-L1+ vesicles / cell
P < 0.0001

Supplementary Data Fig. 9. Rescue experiments in cells with Exo70, PI4KIIα, or MTM1 knockdown. a. RT-qPCR of MTM1 mRNA in cells expressing control shRNA or shRNA targeting the 3' UTR of MTM1. P-value is calculated using one sample t-test. **b.** Localization of PD-L1 and Rab11 in MDA-MB-231 cells stably expressing control shRNA or shRNAs targeting Exo70, PI4KIIα, or MTM1 with or without expression of corresponding rescue plasmids (shExo70 is rescued by Flag-rExo70, PI4KIIα is rescued by Flag-PI4KIIα-WT, and shMTM1 is rescued by mCherry-MTM1). Zoom-in images of the boxed regions are shown in the right column. Scale bar = 10 µm. **c.** Quantification of the Pearson's correlation coefficients for the data in **b**. **d.** Confocal imaging of PD-L1 in cells stably expressing the control shRNA or shRNAs targeting Exo70, PI4KIIα, or MTM1 with their corresponding rescues. LAMP1 is used as a lysosome marker. Zoom-in images of the boxed regions are shown to the right. **e.** Quantification of the LAMP1 positive PD-L1 puncta numbers for the data in **d**. n = 20 cells for all experiments. P values are calculated using a two-sided unpaired t-test unless independently indicated.

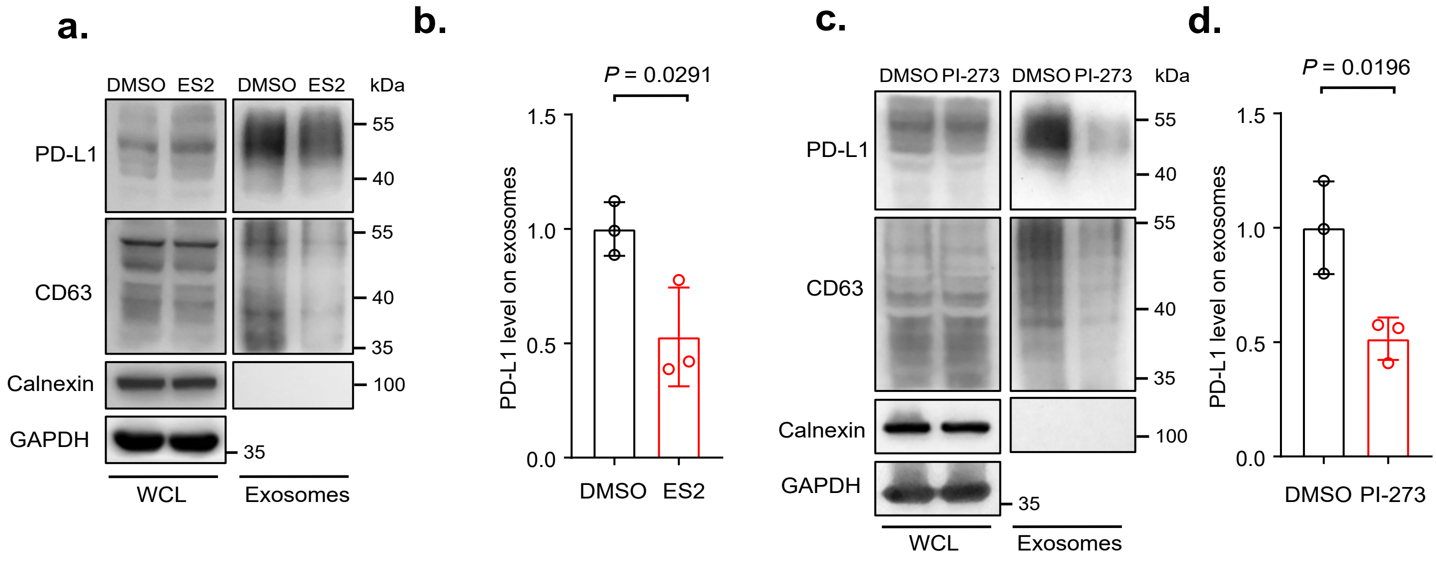
a.
DMSO ES2 DMSO ES2 kDa
PD-L1
CD63
Calnexin
GAPDH
55
40
55
40
35
100
35
WCL
Exosomes
b.
P = 0.0291
PD-L1 level on exosomes
1.5
1.0
0.5
0.0
DMSO ES2
c.
DMSO PI-273 DMSO PI-273 kDa
PD-L1
CD63
Calnexin
GAPDH
55
40
55
40
35
100
35
WCL
Exosomes
d.
P = 0.0196
PD-L1 level on exosomes
1.5
1.0
0.5
0.0
DMSO PI-273

Supplementary Data Fig. 10. Pharmacological inhibition of exosomal secretion of PD-L1. a. Immunoblotting of PD-L1 in the whole cell lysates and exosomes from DMSO or ES2 treated cells. CD63 is used as an exosome marker. Calnexin is used as a negative control. GADPH is used as a loading control. **b.** Quantification of PD-L1 levels in exosomes from DMSO or ES2 treated cells (n=3). **c.** Immunoblotting of PD-L1 in the whole cell lysates and exosomes from DMSO or PI-273 treated cells. **d.** Quantification of PD-L1 levels on exosomes from DMSO or PI-273 treated cells (n=3). The average exosomal PD-L1 level from cells treated with PI-273 is normalized to that of the DMSO-treated control. Data are presented as mean ± s.d. of three independent biological replicates. P values are calculated using a two-sided unpaired t-test. The loading of exosome sample was normalized to the same cell number ($1x10^7$).

Supplementary Data Table 1. Antibodies for IF and WB

Antibody Name	Source	Catalogue No.	Application
Sec8	Santa-Cruz	B-11:sc-514215	WB(1:1000)
Sec10	Santa-Cruz	C-4: sc-514802	WB(1:1000)
Sec15	Guo Lab	N/A	WB(1:1000), IF(1:100)
Exo70	Santa-Cruz	D6:sc-365825	WB(1:1000)
Exo84	Santa-Cruz	H1:sc-515532 Lot: H0918 (Batch number)	WB(1:1000), IF(1:100)
CD63	Abcam	ab134045 ab8219	WB(1:1000) IF(1:250)
CD63-FITC	Herlyn Lab	N/A	IF(1:100)
CD81	CST	(D3N2D) #56039	WB(1:1000)
Calnexin	CST	(C5C9) #2679	WB(1:1000)
EEA1	CST	(C45B10) #3288	WB(1:1000), IF(1:200)
Giantin	Guo Lab	N/A	IF(1:500)
LAMP1	CST	(D2D11) XP® #9091	IF(1:200)
Rab11	Invitrogen	715300	WB(1:1000), IF(1:100)
Rab27a	CST	(D7Z9Q) #69295	IF(1:100)
CoxIV	CST	(3E11) #4850	IF(1:200)
Tom20	Santa-Cruz	F10:sc-17764	WB(1:1000), IF(1:200)
Flag	Sigma-Aldrich	m2 slcc4005	IF(1:100)
PI4KIIα	Santa-Cruz	B5:sc-390026	WB(1:1000), IF(1:100)
PI4KIIIβ	Santa-Cruz BD	E4:sc-166615 (BD) 611817	IF(1:100) WB(1:1000)
PD-L1	CST	(E1L3N®) XP® #13684	WB(1:1000)
PD-L1 (5H1)	Dong Lab	N/A	IF(1:100)
GAPDH	CST	(D16H11) XP® #5174	WB(1:1000)
Syntenin-1	Abcam	ab133267	WB(1:1000)
Tsg101	Santa-Cruz	C-2: sc-7964	WB(1:1000)
DNA-conjugated donkey anti-mouse IgG	Jackson Immuno Research	715-005-150	IF(1:200)

Supplementary Data Table 2. Short hairpin RNAs (shRNAs) Sequences

Gene Name	shRNA sequence
Scramble	CCTAAGGTTAAGTCGCCCTCGCTCGAGCGAGGGCGACTTAACCTTAGGTTTTT (https://www.addgene.org/protocols/plko)
Sec8	#1: CCCTAATTTCTGAGAGAATAA<br>#2: CCCAGAAACAGTTAAGGCAAT
Sec10	#1: TTAGACAGCGTACCAACTTAC<br>#2: AGCAAGCTGATGGAGTTTAAT
Exo70	#1: AACATGGTGTCTATCTTATCA<br>#2: GCTGCAGGAGAATGTTGAGAA
Exo84	#1: GCTGTTCAGCATGTGGATTAT<br>#2: ATGCAGAGAGAGAGCTTATAT
PI4KIIα	#1: GAGCTAGTCTTAGCATTTATA<br>#2: CCTCTTCCTGAGAACACTAAC
PI4KIIIβ	#1: GCAAGAAACACGAAGGATCAT<br>#2: GAGATCCGTTGCCTAGATGAT
MTM1	#1: GCCATTCAAGTAGCAGACAAA<br>#2: TATGAGTGGGAAACGAAATAA<br>#3: CCGCATTAGAGCTCGAAATAA
Rab11a	#1: ATCATGCTGATAGTAACATTG<br>#2: TGGTTTGTCATTCATTGAAA`
