# Supplementary material for: A phosphoinositide switch mediates exocyst recruitment to multivesicular endosomes for exosome secretion
Source: Nat Commun. 2023 Oct 28;14:6883. doi: 10.1038/s41467-023-42661-0 (PMC10613218; doi:10.1038/s41467-023-42661-0)
Supplement: Supplementary file 2 — Description of Additional Supplementary Files [file 41467_2023_42661_MOESM2_ESM.pdf]

Description of Additional Supplementary Files

File name: Supplementary Movie 1

Description: (related to Figure 3) 3D model of a MVE in DMSO treated cells with intraluminal structure.

File name: Supplementary Movie 2

Description: (related to Figure 3) 3D model of a MVE in ES2 treated cells with intraluminal structure.

File name: Supplementary Movie 3

Description: (related to Figure 4). Time-lapse confocal imaging of moving GFP-Exo70^{+} mScarlet-CD63^{+} vesicles. Scale bar = 1 µm.

File name: Supplementary Movie 4

Description: (related to Figure 4). Observation of sfGFP-Exo70^{+} RFP-CD63^{+} vesicles near the plasma membrane by TIRFM. Scale bar = 1 µm.

File name: Supplementary Movie 5

Description: (related to Figure 6). Time-lapse confocal imaging of moving P4C^{+} mScarlet-CD63^{+} vesicles. Scale bar = 1 µm.
