# Supplementary material for: A phosphoinositide switch mediates exocyst recruitment to multivesicular endosomes for exosome secretion
Source: Nat Commun. 2023 Oct 28;14:6883. doi: 10.1038/s41467-023-42661-0 (PMC10613218; doi:10.1038/s41467-023-42661-0)
Supplement: Supplementary file 8 — Reporting Summary [file 41467_2023_42661_MOESM8_ESM.pdf]

nature portfolio

Corresponding author(s): Wei Guo

Last updated by author(s): 2023/10/03

Reporting Summary

Nature Portfolio wishes to improve the reproducibility of the work that we publish. This form provides structure for consistency and transparency in reporting. For further information on Nature Portfolio policies, see our Editorial Policies and the Editorial Policy Checklist.

Statistics

For all statistical analyses, confirm that the following items are present in the figure legend, table legend, main text, or Methods section.

n/a	Confirmed	
☐	☒	The exact sample size (*n*) for each experimental group/condition, given as a discrete number and unit of measurement
☐	☒	A statement on whether measurements were taken from distinct samples or whether the same sample was measured repeatedly
☐	☒	The statistical test(s) used AND whether they are one- or two-sided<br>*Only common tests should be described solely by name; describe more complex techniques in the Methods section.*
☒	☐	A description of all covariates tested
☒	☐	A description of any assumptions or corrections, such as tests of normality and adjustment for multiple comparisons
☐	☒	A full description of the statistical parameters including central tendency (e.g. means) or other basic estimates (e.g. regression coefficient) AND variation (e.g. standard deviation) or associated estimates of uncertainty (e.g. confidence intervals)
☐	☒	For null hypothesis testing, the test statistic (e.g. *F*, *t*, *r*) with confidence intervals, effect sizes, degrees of freedom and *P* value noted<br>*Give P values as exact values whenever suitable.*
☒	☐	For Bayesian analysis, information on the choice of priors and Markov chain Monte Carlo settings
☒	☐	For hierarchical and complex designs, identification of the appropriate level for tests and full reporting of outcomes
☐	☒	Estimates of effect sizes (e.g. Cohen's *d*, Pearson's *r*), indicating how they were calculated

Our web collection on statistics for biologists contains articles on many of the points above.

Software and code

Policy information about availability of computer code

Data collection	Image J (Fiji 2.3.0), NIS-Elements-AR, Nikon's NIS Elements V4.5, NanoSight NTA 3.2, FluorEssence™
Data analysis	statistical analyses were performed with GraphPad Prism 8.3, Gray values for western blotting were analyzed by Image J (Fiji 2.3.0), Fluorescent images were processed in Image J (Fiji 2.3.0) and Nikon's NIS Elements V4.5.

For manuscripts utilizing custom algorithms or software that are central to the research but not yet described in published literature, software must be made available to editors and reviewers. We strongly encourage code deposition in a community repository (e.g. GitHub). See the Nature Portfolio guidelines for submitting code & software for further information.

Data

Policy information about availability of data

All manuscripts must include a data availability statement. This statement should provide the following information, where applicable:

- Accession codes, unique identifiers, or web links for publicly available datasets
- A description of any restrictions on data availability
- For clinical datasets or third party data, please ensure that the statement adheres to our policy

Source data have been provided in Source Data. All other data supporting the findings of this study are available from the corresponding author on reasonable request.

Research involving human participants, their data, or biological material

Policy information about studies with human participants or human data. See also policy information about sex, gender (identity/presentation), and sexual orientation and race, ethnicity and racism.

Reporting on sex and gender	N/A
Reporting on race, ethnicity, or other socially relevant groupings	N/A
Population characteristics	N/A
Recruitment	N/A
Ethics oversight	N/A

Note that full information on the approval of the study protocol must also be provided in the manuscript.

Field-specific reporting

Please select the one below that is the best fit for your research. If you are not sure, read the appropriate sections before making your selection.

☒ Life sciences ☐ Behavioural & social sciences ☐ Ecological, evolutionary & environmental sciences

For a reference copy of the document with all sections, see nature.com/documents/nr-reporting-summary-flat.pdf

Life sciences study design

All studies must disclose on these points even when the disclosure is negative.

Sample size	Samples size for each experiment is indicated in the figures or corresponding supplementary figures. The choice of the sample size is larger than the commonly accepted numbers in the field.
Data exclusions	No exclusion for the data
Replication	Experiments were repeated at least 3 times to ensure reproducibility except otherwise indicated in figure legends.
Randomization	The allocation of cells to different treatments was completely random.
Blinding	Experiments were performed in a blinded fashion whenever possible.

Reporting for specific materials, systems and methods

We require information from authors about some types of materials, experimental systems and methods used in many studies. Here, indicate whether each material, system or method listed is relevant to your study. If you are not sure if a list item applies to your research, read the appropriate section before selecting a response.

Materials & experimental systems

n/a	Involved in the study	
☐	☒	Antibodies
☐	☒	Eukaryotic cell lines
☒	☐	Palaeontology and archaeology
☒	☐	Animals and other organisms
☒	☐	Clinical data
☒	☐	Dual use research of concern
☒	☐	Plants

Methods

n/a	Involved in the study	
☒	☐	ChIP-seq
☒	☐	Flow cytometry
☒	☐	MRI-based neuroimaging

Antibodies

Antibodies used	Sec8 (Santa-Cruz B-11:sc-514215 mAb Mouse, 1:1000 WB)<br>Sec10 Santa-Cruz C-4: sc-514802 mAb Mouse (1:100 WB)<br>Sec15 (mAb Mouse in Lab, 1:1000 WB, 1:100 IF)

Exo70 (Santa-Cruz D6:sc-365825 mAb Mouse, 1:1000 WB) (Proteintech 12014-1-AP polyAb Rabbit, 1:1000 WB)
Exo84 (Santa-Cruz H1:sc-515532 mAb Mouse, 1:1000 WB, Lot: H0918 (Batch number))
CD63 (Abcam ab134045 polyAb Rabbit, 1:1000 WB) (Abcam ab8219 mAb Mouse, 1:100 IF) (CD63-FITC is a gift made from Prof. Meenhard Herlyn and sent by Prof. Michael S. Marks)
CD81 (CST (D3N2D) #56039 Rabbit mAb 1:1000 WB)
Calnexin (CST (C5C9) #2679 Rabbit mAb 1:1000 WB)
EEA1 (CST (C45B10) #3288 Rabbit mAb 1:1000 WB, 1:200 IF)
Giantin (In Guo Lab, 1:100 IF)
LAMP1 (CST (D2D11) XP® #9091 Rabbit mAb, 1:200 IF)
Rab11a (Thermo Invitrogen 715300 polyAb Rabbit, 1:1000 WB, 1:100 IF)
Rab27a (CST (D7Z9Q) #69295 Rabbit mAb 1:100 IF)
CoxIV (CST (3E11) #4850 Rabbit mAb 1:200 IF)
Tom20 (Santa-Cruz F10:sc-17764 mAb Mouse, 1:1000 WB, 1:200 IF)
Flag (Sigma-Aldrich m2 slcc4005 mAb Mouse, 1:100 IF)
PI4KIIα (Santa-Cruz B5:sc-390026 mAb Mouse, 1:1000 WB, 1:100 IF)
PI4KIIIβ (Santa-Cruz E4:sc-166615 mAb Mouse, 1:100 IF) (BD 611817 Mouse 1:1000 WB)
PD-L1 (CST (E1L3N®) XP® #13684 Rabbit mAb 1:1000 WB), (5H1 Dong's Lab mAb Mouse, 1:100 IF)
GAPDH (CST (D16H11) XP® #5174 Rabbit mAb, 1:1000 WB)
Syntenin-1 Abcam ab133267 recombinant Ab (1:1000 WB)
Tsg101 Santa-Cruz C-2: sc-7964 mAb (1:1000 WB)
DNA-conjugated donkey anti-mouse IgG Jackson Immuno Research 715-005-150 (1:200 IF)

Validation

These antibodies were validated for Western Blots or IF by knockdown or knockout experiments:
Sec8 (Santa-Cruz B-11:sc-514215 mAb Mouse, 1:1000 WB)
Sec10 Santa-Cruz C-4: sc-514802 mAb Mouse (1:100 WB)
Sec15 (mAb Mouse in Lab, 1:1000 WB, 1:100 IF)
Exo70 (Santa-Cruz D6:sc-365825 mAb Mouse, 1:1000 WB) (Proteintech 12014-1-AP polyAb Rabbit, 1:1000 WB)
Exo84 (Santa-Cruz H1:sc-515532 mAb Mouse, 1:1000 WB)
CD63 (Abcam ab134045 polyAb Rabbit, 1:1000 WB) (Abcam ab8219 mAb Mouse, 1:100 IF) (CD63-FITC is a gift made from Prof. Meenhard Herlyn and sent by Prof. Michael S. Marks)
CD81 (CST (D3N2D) #56039 Rabbit mAb 1:1000 WB)
Calnexin (CST (C5C9) #2679 Rabbit mAb 1:1000 WB)
EEA1 (CST (C45B10) #3288 Rabbit mAb 1:1000 WB, 1:200 IF)
LAMP1 (CST (D2D11) XP® #9091 Rabbit mAb, 1:200 IF)
Rab11a (Thermo Invitrogen 715300 polyAb Rabbit, 1:1000 WB, 1:100 IF)
Rab27a (CST (D7Z9Q) #69295 Rabbit mAb 1:100 IF)
CoxIV (CST (3E11) #4850 Rabbit mAb 1:200 IF)
Tom20 (Santa-Cruz F10:sc-17764 mAb Mouse, 1:1000 WB, 1:200 IF)
Flag (Sigma-Aldrich m2 slcc4005 mAb Mouse, 1:100 IF)
PI4KIIα (Santa-Cruz B5:sc-390026 mAb Mouse, 1:1000 WB, 1:100 IF)
PI4KIIIβ (Santa-Cruz E4:sc-166615 mAb Mouse, 1:100 IF) (BD 611817 Mouse 1:1000 WB)
PD-L1 (CST (E1L3N®) XP® #13684 Rabbit mAb 1:1000 WB), (5H1 Dong's Lab mAb Mouse, 1:100 IF)
GAPDH (CST (D16H11) XP® #5174 Rabbit mAb, 1:1000 WB)
Syntenin-1 Abcam ab133267 recombinant Ab (1:1000 WB)
Tsg101 Santa-Cruz C-2: sc-7964 mAb (1:1000 WB)

These antibodies are validated for IF only:
Giantin (In Guo Lab, 1:100 IF)
DNA-conjugated donkey anti-mouse IgG Jackson Immuno Research 715-005-150 (1:200 IF)

Detailed validation statement for antibody is provided on the manufacture's websites. Antibodies were further validated by using positive and negative controls in our studies including those not obtained from manufactures.

Eukaryotic cell lines

Policy information about cell lines and Sex and Gender in Research

Cell line source(s)

COS-7 and PANC-1 were obtained from ATCC. MDA-MB-231 was from Georgetown Lombardi Comprehensive Cancer Center (LCCC). NMuMG was from Dr. Ian G. Macara

Authentication

All of the cell lines used were authenticated by STR profiling.

Mycoplasma contamination

Mycoplasma testing was regularly conducted to assure that all cells used were mycoplasma free.

Commonly misidentified lines
(See ICLAC register)

No commonly misidentified cell lines were used for this study.
