# Supplementary material for: A phosphoinositide switch mediates exocyst recruitment to multivesicular endosomes for exosome secretion
Source: Nat Commun. 2023 Oct 28;14:6883. doi: 10.1038/s41467-023-42661-0 (PMC10613218; doi:10.1038/s41467-023-42661-0)

Figure 1b

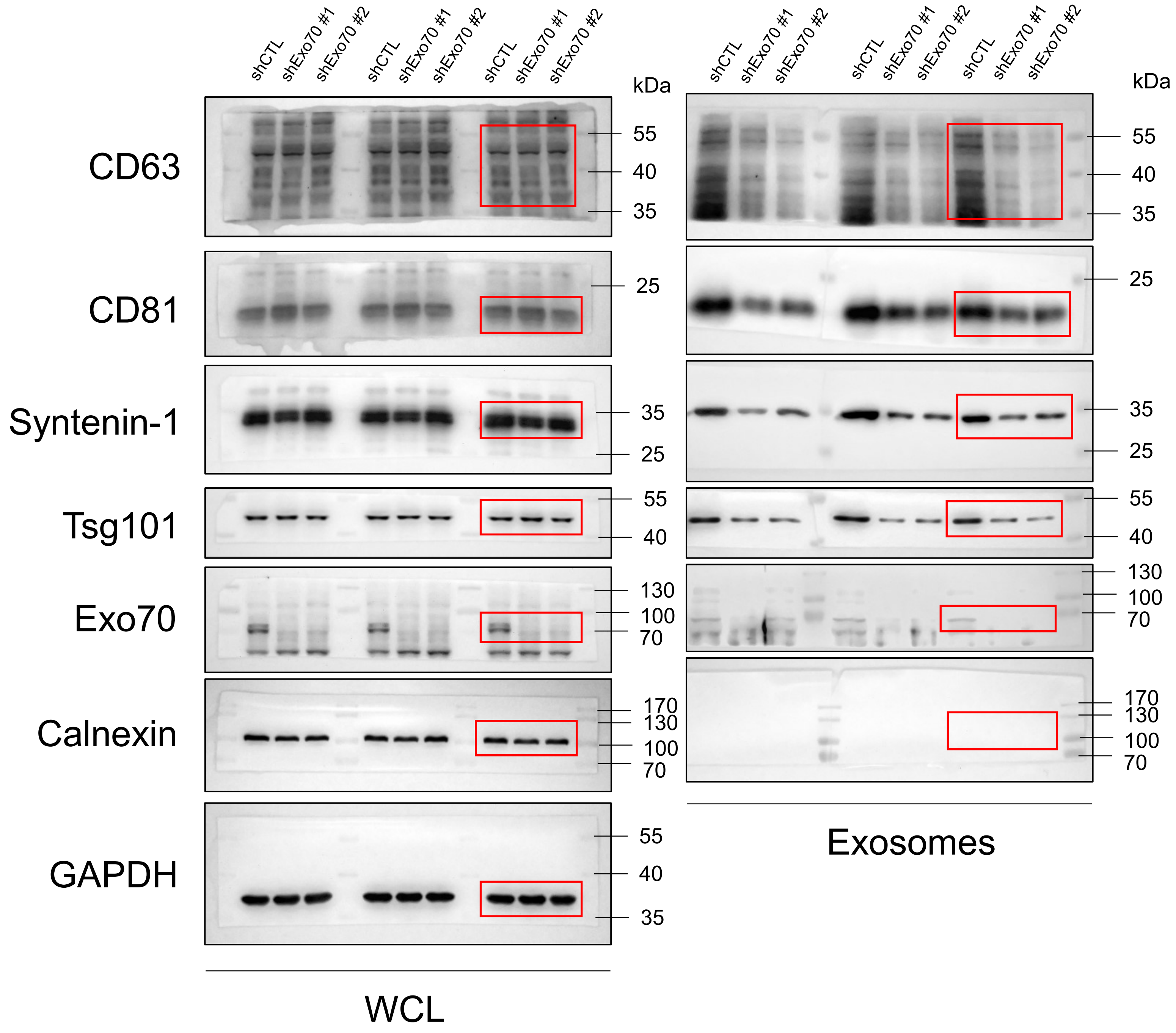
shCTL
shExo70 #1
shExo70 #2
shCTL
shExo70 #1
shExo70 #2
shCTL
shExo70 #1
shExo70 #2
kDa
CD63
55
40
35
CD81
25
Syntenin-1
35
25
Tsg101
55
40
Exo70
130
100
70
Calnexin
170
130
100
70
GAPDH
55
40
35
WCL
shCTL
shExo70 #1
shExo70 #2
shCTL
shExo70 #1
shExo70 #2
shCTL
shExo70 #1
shExo70 #2
kDa
55
40
35
25
35
25
55
40
130
100
70
170
130
100
70
Exosomes

Figure 1e

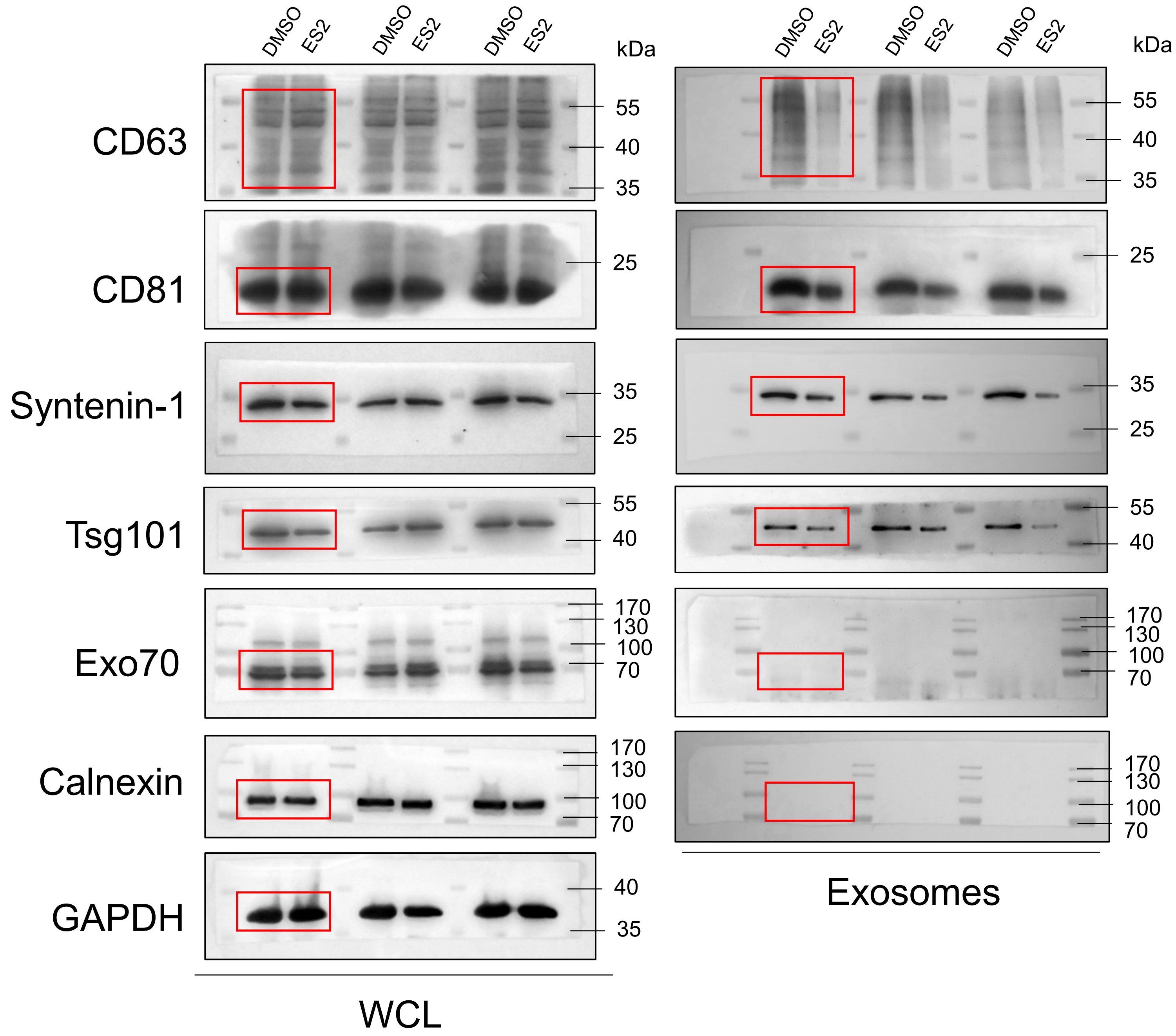

DMSO
ES2
DMSO
ES2
DMSO
ES2
kDa
CD63
CD81
Syntenin-1
Tsg101
Exo70
Calnexin
GAPDH
55
40
35
25
35
25
55
40
170
130
100
70
170
130
100
70
40
35
WCL
DMSO
ES2
DMSO
ES2
DMSO
ES2
kDa
55
40
35
25
35
25
55
40
170
130
100
70
170
130
100
70
Exosomes

Supplemnetary Figure 1e

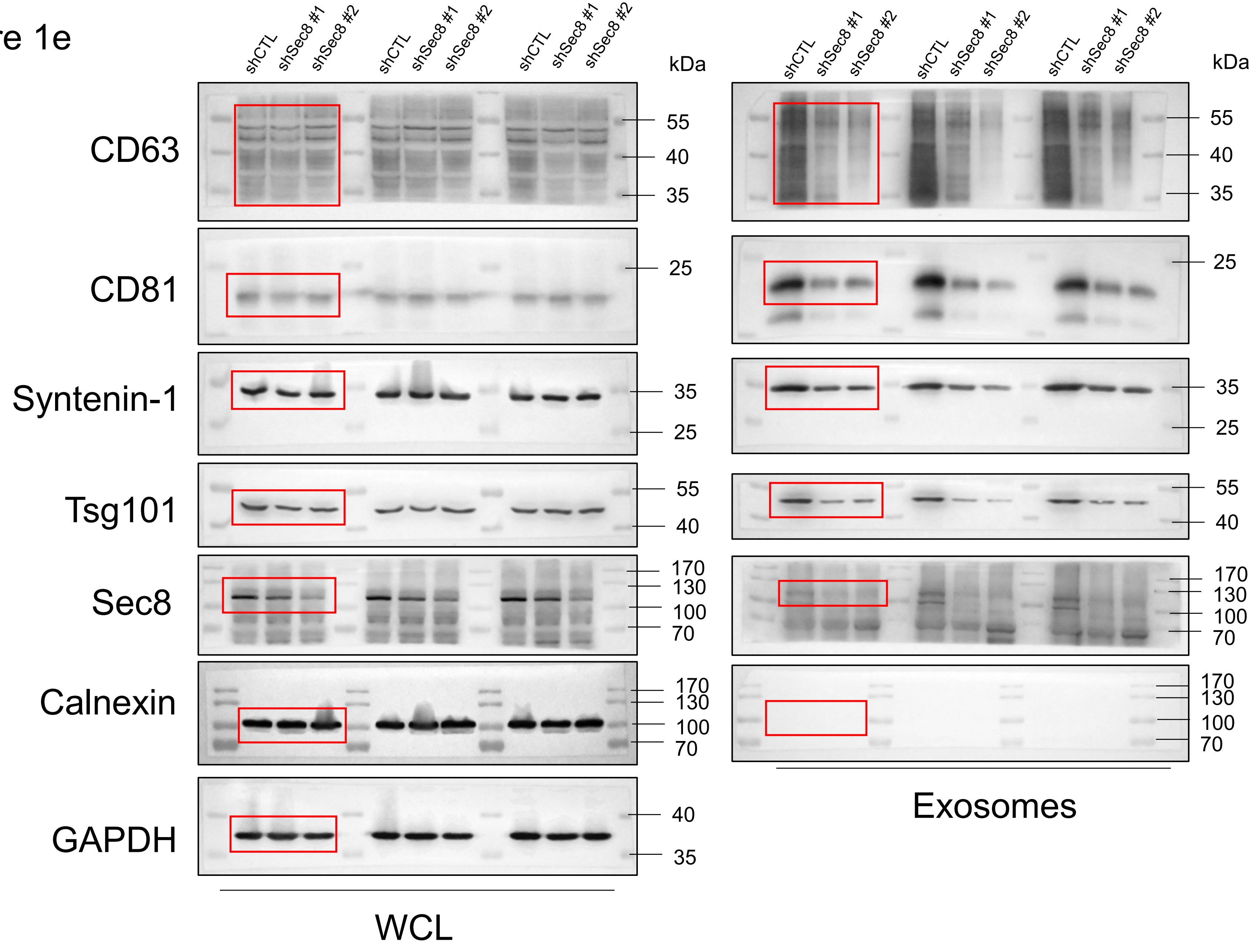

Supplementary Figure 1g

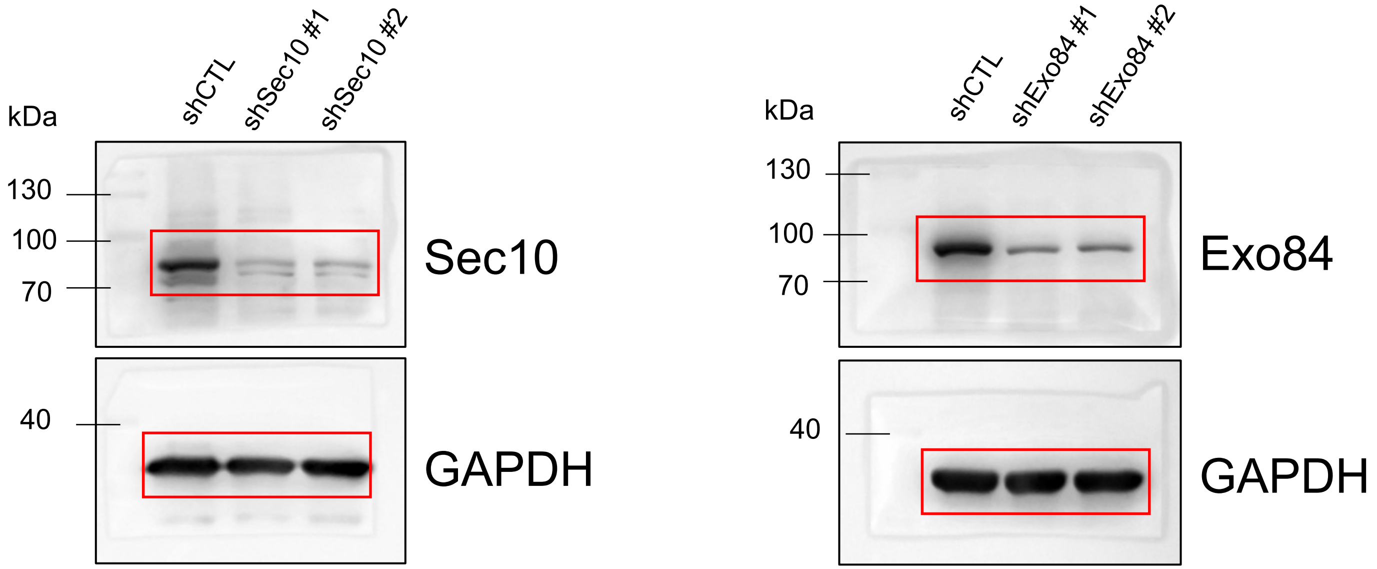

Supplementary Figure 2a

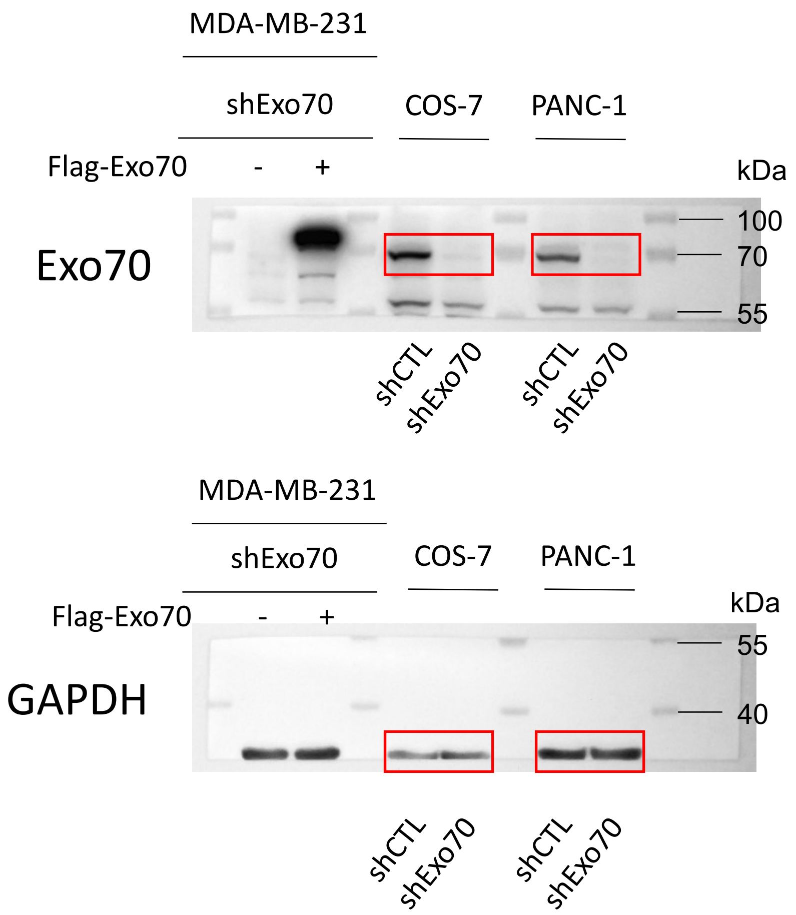

Supplementary Figure 7d

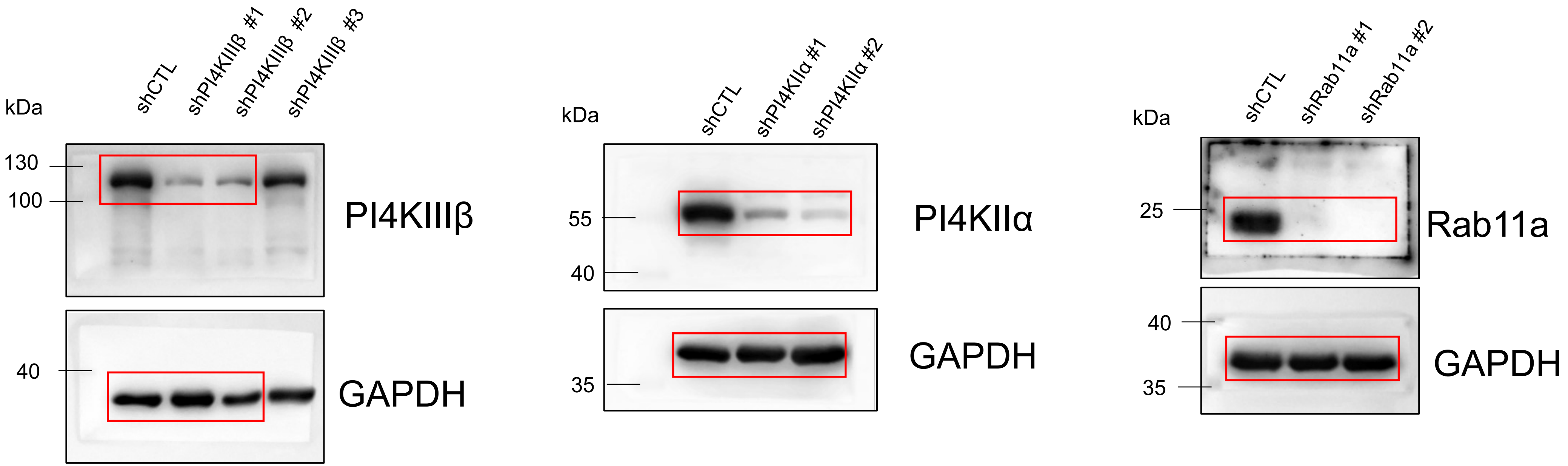

Supplementary Figure 10a

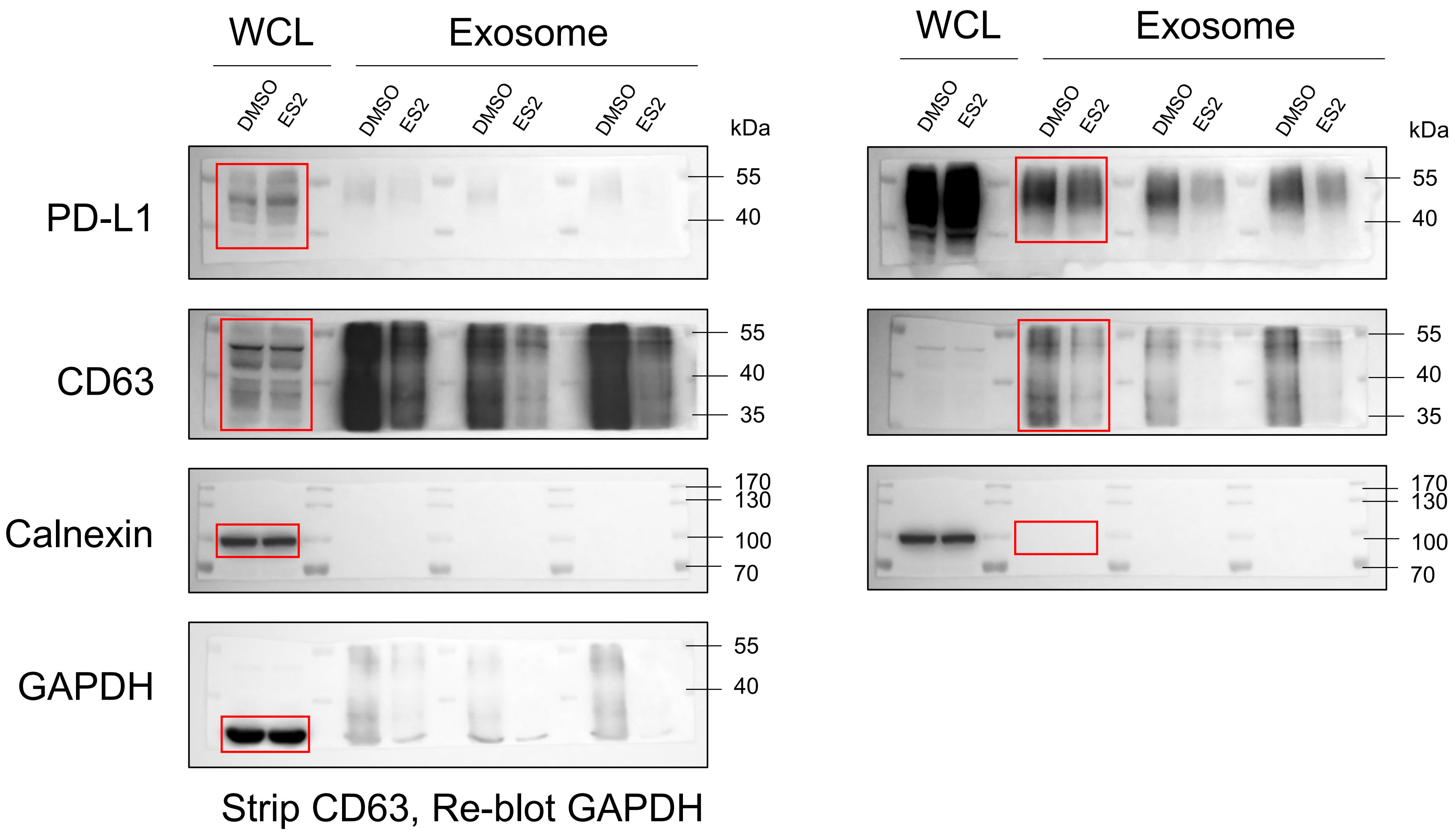

Supplementary Figure 10c

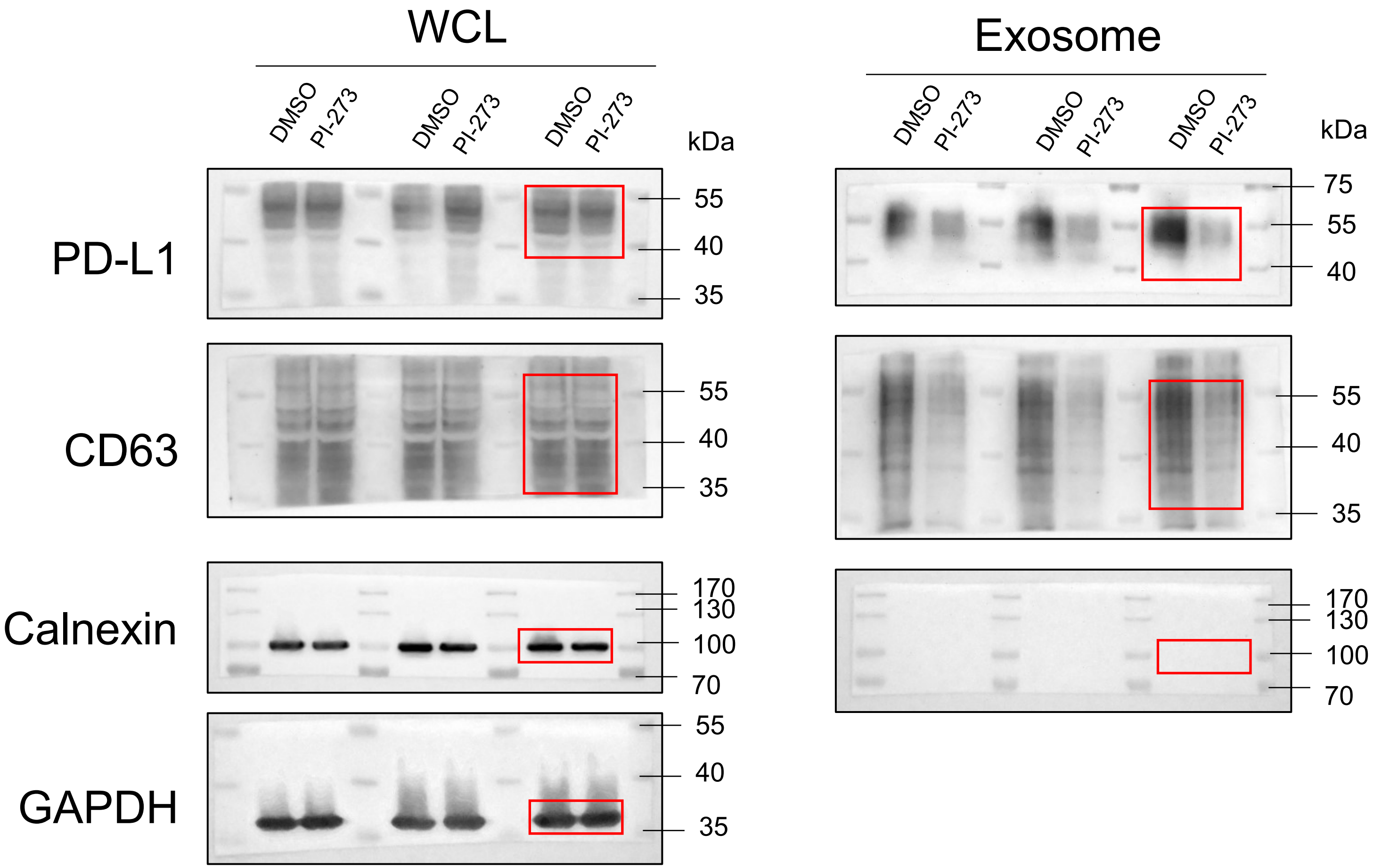

Supplement: Supplementary file 9 — Source Data [file 41467_2023_42661_MOESM9_ESM.zip › 357425_4_data_set_8139057_s283r3/Source_Data_2.pdf]
